# Supplementary material for: Multi-trait analysis characterizes the genetics of thyroid function and identifies causal associations with clinical implications
Source: Nat Commun. 2024 Jan 30;15:888. doi: 10.1038/s41467-024-44701-9 (PMC10828500; doi:10.1038/s41467-024-44701-9)

Multi-trait analysis characterizes the genetics of thyroid function and identifies causal associations with clinical implications

Sterenborg *et al.*

SUPPLEMENTARY INFORMATION

Table of Contents

Supplementary Note: Extended acknowledgements and ethics approvals............................3

Supplementary Figure 1: Overview of sample sizes per cohort per thyroid function trait.....13

Supplementary Figure 2: Mirrored Manhattan plot FT3 and TT3 14

Supplementary Figure 3: Mirrored Manhattan plot FT3/FT4 ratio and TT3/FT4 ratio 15

Supplementary Figure 4: Mirrored Manhattan plot high TSH and low TSH...........................16

Supplementary Figure 5: Beta-beta plots of TSH and FT4 index variants versus previous GWAS 17

Supplementary Figure 6: Quantile-quantile plots for all thyroid function traits 18

Supplementary Figure 7: Fine-mapping results..20

Supplementary Figure 8. Colocalizations of high and low TSH in the tissues of the HTP axis 21

Supplementary Figure 9. Top 10 canonical pathways for TSH, FT4, FT3/FT4 ratio and low TSH. 22

Supplementary Figure 10: Tissue expression analysis using MAGMA 23

Supplementary Figure 11. Risk of thyroid cancer in Icelandic individuals by various thyroid function parameter polygenic risk scores...27

Supplementary Figure 12. Mendelian Randomization forest plot TSH vs thyroid cancer 28

Supplementary Figure 13. Mendelian randomization effect-effect plots on thyroid traits and non-medullary thyroid cancer 29

Supplementary Note: Extended acknowledgements and ethics approvals

The Genotype-Tissue Expression (GTEx) Project was supported by the Common Fund of the Office of the Director of the National Institutes of Health, and by NCI, NHGRI, NHLBI, NIDA, NIMH, and NINDS.

Adigen
Novo Nordisk Foundation Center for Basic Metabolic Research is an independent Research Center, based at the University of Copenhagen, Denmark and partially funded by an unconditional donation from the Novo Nordisk Foundation (www.cbmr.ku.dk) (Grant number NNF18CC0034900). The study was approved by the ethical committees for Frederiksberg and Copenhagen and was in accordance with the guidelines of the second Helsinki Declaration. All participants signed a written consent before participating in the study.

Amish
The Amish study was supported by NIH grants U01 HL088120, R01AG18728, R01 HL69313, and U01 GM074518. The Amish study was approved by the University of Maryland Institutional Review Board.

ARIC
The Atherosclerosis Risk in Communities study has been funded in whole or in part with Federal funds from the National Heart, Lung, and Blood Institute, National Institutes of Health, Department of Health and Human Services, under Contract nos. (75N92022D00001, 75N92022D00002, 75N92022D00003, 75N92022D00004, 75N92022D00005). The authors thank the staff and participants of the ARIC study for their important contributions. Funding was also supported by R01HL087641 and R01HL086694; National Human Genome Research Institute contract U01HG004402; and National Institutes of Health contract HHSN268200625226C. Infrastructure was partly supported by Grant Number UL1RR025005, a component of the National Institutes of Health and NIH Roadmap for Medical Research. The ARIC study was approved by the Institutional Review Board of each ARIC site (IRB No.: 12998/CR517): University of North Carolina at Chapel Hill, Chapel Hill, NC; Wake Forest University, Winston-Salem, NC; Johns Hopkins University, Baltimore, MD; University of Minnesota, Minneapolis, MN; and University of Mississippi Medical Center, Jackson, MS.

BHS
The 1994/1995 Busselton Health Survey was supported and funded by the Health Promotion Foundation of Western Australia. The study has received ethics approval from the University of Western Australia Human Research Ethics Committee (Number RA/4/1/2203).

BLSA
The BLSA was supported by the Intramural Research Program of the NIH, National Institute on Aging. This work utilized the computational resources of the NIH HPC Biowulf cluster. (http://hpc.nih.gov) The BLSA study protocol for both studies were reviewed and approved by the Internal Review Board of the National Institute for Environmental Health Sciences (NIEHS) and all participants provided written informed consent.

BLTS
The plasma samples were collected in the context of the Brisbane Longitudinal Twin Study 1992–2016, supported by grants from Australian National Health and Medical Research Council (NHMRC; project grant 1031119; NGM) and Australian Research Council (DP1093900; NGM; MJW). The study was approved by the Human Research Ethics committee of the Queensland Institute of Medical Research

CHS
This research was supported by contracts 75N92021D00006, HHSN268201200036C, HHSN268200800007C, HHSN268201800001C, N01HC55222, N01HC85079, N01HC85080, N01HC85081, N01HC85082, N01HC85083, N01HC85086, 75N92021D00006, and grants U01HL080295, R01HL103612, R01HL120393 and U01HL130114 from the National Heart, Lung, and Blood Institute (NHLBI), with additional contribution from the National Institute of Neurological Disorders and Stroke (NINDS). Additional support was provided by R01AG023629 and R01AG032317 from the National Institute on Aging (NIA). Support for the Cardiovascular Health Study Whole Genome Study was provided by NHLBI grant R01HL087652. Additional support for infrastructure was provided by R01HL105756 and additional genotyping among the African-American cohort was supported in part by R01HL085251. DNA handling and genotyping at Cedars-Sinai Medical Center was supported in part by National Center for Research Resources grant UL1RR033176, now at the National Center for Advancing Translational Technologies CTSI grant UL1TR001881; in addition to the National Institute of Diabetes and Digestive and Kidney Diseases grant DK063491 to the Southern California Diabetes Endocrinology Research Center. BNH was supported by a National Heart, Lung and Blood Institute grant T32HL007828. A full list of principal CHS investigators and institutions can be found at CHS-NHLBI.org. The content is solely the responsibility of the authors and does not necessarily represent the official views of the National Institutes of Health. The institutional review boards of all four sites and the coordinating center at the University of Washington in Seattle approved the study. All participants gave informed consent.

CHRIS
Full acknowledgements for the CHRIS study are reported at http://translational-medicine.biomedcentral.com/articles/10.1186/s12967-015-0704-9#Declarations. The CHRIS study was funded by the Department of Innovation, Research, and University of the Autonomous Province of Bolzano-South Tyrol. The CHRIS study was approved by the Ethics Committee of the Healthcare System of the Autonomous Province of Bolzano/Bozen.

CROATIA Split – Korcula – Vis
We would like to acknowledge the staff of several institutions in Croatia that supported the field work, including but not limited to The University of Split and Zagreb Medical Schools, Institute for Anthropological Research in Zagreb and Croatian Institute for Public Health. Croatian Science Foundation project "Regulation of Thyroid and Parathyroid Function and Blood Calcium Homeostasis" (No. 2593). Medical Research Council (UK). European Commission Framework 6 project EUROSPAN (Contract No. LSHG-CT-2006018947). Republic of Croatia Ministry of Science, Education and Sports research grant (216-1080315-0302). CEKOM (Ministry of Economy, Entrepreneurship and Crafts). Croatian National Centre of Research Excellence in Personalized Healthcare grant (number KK.01.1.1.01.0010). Centre of

Competence in Molecular Diagnostics (KK.01.2.2.03.0006). CH is supported by an MRC University Unit Programme Grant MC_UU_00007/10 (QTL in Health and Disease).
Ethics approval statement: Committee of the University of Split School of Medicine (2181-198-03-04-14-0031 and 2181-198-03-04-19-0022 and 2181-198-03-04/10-11-0008)

DeCODE
The study was approved by the Icelandic National Bioethics Committee (VSN-17-204).

EPIC-Norfolk
We are grateful to all the participants who have been part of the project and to the many members of the study teams at the University of Cambridge who have enabled this research. The EPIC-Norfolk study (https://doi.org/10.22025/2019.10.105.00004) has received funding from the Medical Research Council (MR/N003284/1 and MC-UU_12015/1) and Cancer Research UK (C864/A14136). The EPIC-Norfolk study was approved by the Norfolk Research Ethics Committee (ref. 05/Q0101/191) and all participants gave their written consent before entering the study.

Fenland
We are grateful to all the participants who have been part of the project and to the many members of the study teams at the University of Cambridge who have enabled this research. The EPIC-Norfolk study (https://doi.org/10.22025/2019.10.105.00004) has received funding from the Medical Research Council (MR/N003284/1 and MC-UU_12015/1) and Cancer Research UK (C864/A14136). The Fenland study was approved by the National Health Service (NHS) Health Research Authority Research Ethics Committee (NRES Committee – East of England Cambridge Central, ref. 04/Q0108/19), and all participants provided written informed consent.

FHS
All participants from the Framingham Heart Study gave informed consent for participation in this study and for the collection of plasma and DNA for analysis. The FHS study protocol was approved by Boston Medical Center.

GARP
We thank all study participants of the GARP study. Leiden University Medical Centre, Pfizer Groton, Connecticut, USA and the Dutch Arthritis Society have supported the GARP study. Furthermore, the research leading to these results has received funding from the Biobanking and BioMolecular resources Research Infrastructure the Netherlands (BBMRI-NL) (complementation project CP2013-84), and the Dutch Arthritis Society (DAF_10_1-402). The GARP study was approved by the medical ethics committee of the Leiden University Medical Center (P76-98) and informed consent was obtained from all participants. We are indebted to drs. N. Riyazi, J. Bijsterbosch, H.M. Kroon and I. Watt for collection of data.

GCKD
The GCKD study was/is supported by the German Federal Ministry of Education and Research (Bundesministerium für Bildung und Forschung, FKZ 01ER 0804, 01ER 0818, 01ER 0819, 01ER 0820, and 01ER 0821), the KfH Foundation for Preventive Medicine. Unregistered grants to support the study were provided by corporate sponsors (listed at

gckd.org). Genotyping in GCKD was supported by Bayer Pharma AG. We are grateful for the willingness of the patients to participate in the GCKD study. The enormous effort of the study personnel of the various regional centers is highly appreciated. We thank the large number of nephrologists who provide routine care for the patients and collaborate with the GCKD study. The GCKD Study was approved by local ethic committees and registered in the national registry for clinical studies (DRKS 00003971).

GESUS
We thank the study participants for generously taking their time to participate in the study. We thank the study staff for their hard work and dedication. The study was approved by the regional ethical committee (SJ-113, SJ-114). It was approved by the Danish Data Protection Agency (REG-27-2014) and follows GDPR rules. Written informed consent was obtained from all participants. The study followed the principles of the Declaration of Helsinki. Funding: Johan and Lise Boserup Foundation; TrygFonden; Det Kommunale Momsfond; Johannes Fog's Foundation; Region Zealand; Region Zealand Foundation; Naestved Hospital; Naestved Hospital Foundation; The Danish Health Authority; Karen Elise Jensen foundation; Edith and Henrik Henriksen's mindelegat; Danish Agency for Science, Technology and Innovation; the Research Council at Rigshospitalet. The genotyping was supported as part of DANFIB by The John and Birthe Meyer Foundation, The Research Foundation at Rigshospitalet, Villadsen Family Foundation, The Arvid Nilsson Foundation, and The Hallas-Møller Emerging Investigator Novo Nordisk (NNF17OC0031204). Christina Ellervik is partly funded by the Laboratory Medicine Endowment Fund of Boston Children's Hospital.

GODARTS
We are grateful to all participants in this study, the general practitioners, the Scottish School of Primary Care for their help in recruiting participants, and to the whole team, which includes interviewers, computer and laboratory technicians, clerical workers, research scientists, volunteers, managers, receptionists and nurses. The study complies with the Declaration of Helsinki. Data provision and linkage was carried out by the Health Informatics Centre, University of Dundee (HIC: https://www.dundee.ac.uk/hic) with analysis of anonymised data performed in an International Organization for Standardisation 27001 – and Scottish Government accredited, secure safe haven. HIC standard operating procedures have been reviewed and approved by the NHS East of Scotland Research Ethics Services, and consent for this study was obtained from the NHS Fife Caldicott Guardian. We would also like to acknowledge NHS Tayside the original data owner. Genomic data QC and analyses were undertaken using High Performance Computing hosted by the School of Life Sciences, University of Dundee https://www.lifesci.dundee.ac.uk/high-performance-computing-0 GoDARTS is funded and supported by the Wellcome Trust, Tenovus Scotland and Diabetes UK grants. SHARE is NHS Scotland Research (NRS) infrastructure initiative and if funded by the Chief Scientists Office of the Scottish Government. Additional Funding and initiation of the spare blood retention at NHS Tayside was supported by the Wellcome Trust Biomedical Resource Award Number 099177/Z/12/Z.
Ethics committee approval for GoDARTS was provided Tayside Medical Ethics Committee 053/04. Ethics committee SHARE: East of Scotland NHS REC 13/ES/0020

HBCS
We thank all study participants as well as everybody involved in the Helsinki Birth Cohort

Study. Helsinki Birth Cohort Study has been supported by grants from the Academy of Finland, the Finnish Diabetes Research Society, Folkhälsan Research Foundation, Novo Nordisk Foundation, Finska Läkaresällskapet, Juho Vainio Foundation, Signe and Ane Gyllenberg Foundation, University of Helsinki, Ministry of Education, Jalmari ja Rauha Ahokas foundation, Emil Aaltonen Foundation, and Yrjö Jahnsson foundation.
Research plan of the HBCS was approved by the Institutional Review Board of the National Public Health Institute and all participants have signed an informed consent.

Health2006
The Health 2006 was financially supported by grants from the Velux Foundation; The Danish Medical Research Council, Danish Agency for Science, Technology and Innovation; The Aase and Ejner Danielsens Foundation; ALK-Abello A/S, Hørsholm, Denmark, and Research Centre for Prevention and Health, the Capital Region of Denmark. A written informed consent form was obtained from all participants and the study was approved by the Ethical Committee of Copenhagen County (KA-20060011) and the Danish Data Protection Agency.

Health2008
Health 2008 was supported by the Timber Merchant Vilhelm Bang's Foundation, the Danish Heart Foundation (Grant number 07-10-R61-A1754-B838-22392F), and the Health Insurance Foundation (Helsefonden) (Grant number 2012B233). The study was approved by the Ethics Committee of the Copenhagen Region (KA-20060011) and all participants provided written informed consent.

HUNT
The HUNT Study is a collaboration between the HUNT Research Centre (Faculty of Medicine and Health Sciences, NTNU, Norwegian University of Science and Technology), Trøndelag County Council, Central Norway Regional Health Authority, and the Norwegian Institute of Public Health. The genotyping in HUNT was financed by the National Institutes of Health; University of Michigan; the Research Council of Norway; the Liaison Committee for Education, Research and Innovation in Central Norway; and the Joint Research Committee between St Olav's hospital and the Faculty of Medicine and Health Sciences, NTNU. WZ was supported by the National Human Genome Research Institute of the National Institutes of Health under award number T32HG010464. The K.G. Jebsen Center for Genetic Epidemiology is funded by Stiftelsen Kristian Gerhard Jebsen; Faculty of Medicine and Health Sciences, NTNU; The Liaison Committee for education, research and innovation in Central Norway; and the Joint Research Committee between St. Olavs Hospital and the Faculty of Medicine and Health Sciences, NTNU. Participation in HUNT is based on informed consent, and the study has been approved by the Norwegian Data Protection Authority and the Regional Committee for Medical and Health Research Ethics in Central Norway.

InCHIANTI
The InCHIANTI study baseline (1998-2000) was supported as a "targeted project" (ICS110.1/RF97.71) by the Italian Ministry of Health and in part by the U.S. National Institute on Aging (Contracts: 263 MD 9164 and 263 MD 821336));supported in part by the Intramural Research Program of the National Institute on Aging, National Institutes of Health, Baltimore, Maryland. This work utilized the computational resources of the NIH HPC Biowulf cluster. (http://hpc.nih.gov). The study protocol was approved by the Italian

National Institute of Research and Care of Aging Institutional Review and by the Internal Review Board of the National Institute for Environmental Health Sciences (NIEHS)

Inter99

The Inter99 was initiated by Torben Jørgensen (PI), Knut Borch-Johnsen (co-PI), Hans Ibsen and Troels F. Thomsen. The steering committee comprises the former two and Charlotta Pisinger. The study was financially supported by research grants from the Danish Research Council, the Danish Centre for Health Technology Assessment, Novo Nordisk Inc., Research Foundation of Copenhagen County, Ministry of Internal Affairs and Health, the Danish Heart Foundation, the Danish Pharmaceutical Association, the Augustinus Foundation, the Ib Henriksen Foundation, the Becket Foundation, and the Danish Diabetes Association. All participants gave a written consent before taking part in the study. The study was approved by the local ethical committee (KA 98 155).

KORA

The KORA study was initiated and financed by the Helmholtz Zentrum München – German Research Center for Environmental Health, which is funded by the German Federal Ministry of Education and Research (BMBF) and by the State of Bavaria. Data collection in the KORA study is done in cooperation with the University Hospital of Augsburg. Furthermore, KORA research was supported within the Munich Center of Health Sciences (MC Health), Ludwig-Maximilians-Universität, as part of LMUinnovativ. Funded by the Bavarian State Ministry of Health and Care through the research project DigiMed Bayern (www.digimed-bayern.de). We thank all participants for their long-term commitment to the KORA study, the staff for data collection and research data management and the members of the KORA Study Group (https://www.helmholtz-munich.de/en/epi/cohort/kora) who are responsible for the design and conduct of the study. The KORA cohort ethical approval was granted by the ethics committee of the Bavarian Medical Association (REC reference number F4: #06068). This covers consent for the use of biological material, including genetics. The KORA data protection procedures were approved by the responsible data protection officer of the Helmholtz Zentrum München.

LBC

We thank the cohort participants and team members who contributed to these studies. Phenotype collection in the Lothian Birth Cohort 1921 was supported by the UK's Biotechnology and Biological Sciences Research Council (BBSRC), The Royal Society, and The Chief Scientist Office of the Scottish Government. LBC1936 is supported by the BBSRC, and the Economic and Social Research Council [BB/W008793/1], Age UK (Disconnected Mind project), and the University of Edinburgh. Genotyping of the cohorts was funded by the BBSRC (BB/F019394/1). Ethics permission for the LBC1936 was obtained from the Multi-Centre Research Ethics Committee for Scotland (MREC/01/0/56) and the Lothian Research Ethics Committee (LREC/2003/2/29). Ethics permission for the LBC1921 was obtained from the Lothian Research Ethics Committee (LREC/1998/4/183). All persons gave their informed consent prior to their inclusion in the study. We gratefully acknowledge the contribution of LBC co-author Professor John M. Starr, who died prior to the publication of this manuscript.

Leiden Longevity Study

The Leiden Longevity Study has received funding from the European Union's Seventh Framework Programme (FP7/2007-2011) under grant agreement number 259679. This study was financially supported by the Innovation-Oriented Research Program on Genomics (SenterNovem IGE05007), the Centre for Medical Systems Biology and the Netherlands Consortium for Healthy Ageing (grant 050-060-810), all in the framework of the Netherlands Genomics Initiative, Netherlands Organization for Scientific Research (NWO), by Unilever Colworth and by BBMRI-NL, a Research Infrastructure financed by the Dutch government (NWO 184.021.007). Dina Vojinovic is supported by Medical Delta, scientific program METABODELTA: Metabolomics for clinical advances in the Medical Delta and the Netherlands Consortium of Dementia Cohorts (NCDC). NCDC receives funding in the context of Deltaplan Dementie from ZonMW Memorabel (projectnr 73305095005) and Alzheimer Nederland. This work is supported by the research programme VOILA with project number 457001001, which is (partly) financed by ZonMw (The Netherlands Organization for Health Research and Development). The Leiden Longevity Study was approved by the medical ethical committee of the Leiden University Medical Center.

LifeLines

The authors acknowledge the services of the Lifelines Cohort Study, the contributing research centers delivering data to Lifelines, and all the study participants. The Lifelines Biobank initiative has been made possible by subsidy from the Dutch Ministry of Health, Welfare and Sport, the Dutch Ministry of Economic Affairs, the University Medical Center Groningen (UMCG the Netherlands), University Groningen and the Northern Provinces of the Netherlands. There was no funding for this manuscript. Ethical approval for the Lifelines Cohort Study was provided by the medical ethical committee of the University Medical Center Groningen, the Netherlands. Informed consent was obtained from all individual participants included in the study.

Michigan Genomics Initiative

The authors acknowledge the Michigan Genomics Initiative participants, Precision Health at the University of Michigan, and the University of Michigan Medical School Data Office for Clinical and Translational Research for providing data storage, management, processing, and distribution services. Analysis of the Michigan Genomics Initiative dataset was approved by the Institutional Review Board at the University of Michigan

Nijmegen Biomedical Study

The Nijmegen Biomedical Study is a population-based survey conducted at the Department for Health Evidence and the Department of Laboratory Medicine of the Radboud university medical center. Principal investigators of the Nijmegen Biomedical Study are L.A.L.M. Kiemeney, A.L.M. Verbeek, D.W. Swinkels en B. Franke. Approval to conduct the NBS was obtained from the Radboud university medical center Institutional Review Board. All participants gave written informed consent.

PROSPER

The PROSPER study was supported by an investigator initiated grant obtained from Bristol-Myers Squibb. Prof. Dr. J. W. Jukema is an Established Clinical Investigator of the Netherlands Heart Foundation (grant 2001 D 032). Support for genotyping was provided by

the seventh framework program of the European commission (grant 223004) and by the Netherlands Genomics Initiative (Netherlands Consortium for Healthy Aging grant 050-060-810). The study was approved by the institutional ethics review boards of centers of Cork University (Ireland), Glasgow University (Scotland) and Leiden University Medical Center (the Netherlands) and all participants gave written informed consent.

Regeneron/Geisinger DiscovEHR (MyCode)

The genomic data are stored in a secure database and made available to investigators, contingent on approval by the MyCode Governing Board. Internal oversight of the GHS biobanking program is provided by the MyCode Governing Board. An additional layer of independent oversight is provided by an Ethics Advisory Council comprising external experts in genetics and ethics as well as members from the local community who are MyCode participants and a separate Return of Results Oversight Committee comprising experts in genetics, clinical medicine, and bioethics.

Rotterdam Study

The Rotterdam Study2 is funded by Erasmus Medical Center and Erasmus University, Rotterdam, Netherlands Organization for the Health Research and Development (ZonMw), the Research Institute for Diseases in the Elderly (RIDE), the Ministry of Education, Culture and Science, the Ministry for Health, Welfare and Sports, the European Commission (DG XII), and the Municipality of Rotterdam. The authors are grateful to the study participants, the staff from the Rotterdam Study and the participating general practitioners and pharmacists. The Rotterdam Study has been approved by the Medical Ethics Committee of the Erasmus MC (registration number MEC 02.1015) and by the Dutch Ministry of Health, Welfare and Sport (Population Screening Act WBO, license number 1071272-159521-PG). The Rotterdam Study has been entered in the Netherlands National Trial Register (NTR; http://www.trialregister.nl) and in the WHO International Clinical Trials Registry Platform (ICTRP; http://www.who.int/ictrp/network/primary/en/) under shared catalogue number NTR6831. All participants provided written informed consent to participate in the study and to have their information obtained from treating physicians. The generation and management of GWAS genotype data for the Rotterdam Study (RS I, RS II, RS III) was executed by the Human Genotyping Facility of the Genetic Laboratory of the Department of Internal Medicine, Erasmus MC, Rotterdam, The Netherlands. The GWAS datasets are supported by the Netherlands Organisation of Scientific Research NWO Investments (nr. 175.010.2005.011, 911-03-012), the Genetic Laboratory of the Department of Internal Medicine, Erasmus MC, the Research Institute for Diseases in the Elderly (014-93-015; RIDE2), the Netherlands Genomics Initiative (NGI)/Netherlands Organisation for Scientific Research (NWO) Netherlands Consortium for Healthy Aging (NCHA), project nr. 050-060-810. We thank Pascal Arp, Mila Jhamai, Marijn Verkerk, Lizbeth Herrera and Marjolein Peters, MSc, and Carolina Medina-Gomez, MSc, for their help in creating the GWAS database, and Karol Estrada, PhD, Yurii Aulchenko, PhD, and Carolina Medina-Gomez, MSc, for the creation and analysis of imputed data. This study makes use of sequence reference data generated by the UK10K Consortium, derived from samples from the ALSPAC and TwinsUK datasets. A full list of the investigators who contributed to the generation of the data is available from www.UK10K.org. Funding for UK10K was provided by the Wellcome Trust under award WT091310. We thank Jie Huang at the Wellcome Trust's Sanger Institute, at Hinxton, U.K. for the creation of imputed data, with the support of

Marijn Verkerk, Carolina Medina-Gomez, MSc, and Anis Abuseiris and their input for the analysis setup. This study makes use of an extended dataset of RS II and RS III samples based on Illumina Omni 2.5 and 5.0 GWAS genotype data, imputed to 1KG using the two-phase imputation method3. This dataset was funded by the Genetic Laboratory of the Department of Internal Medicine, the department of Forensic Molecular Biology, and the department of Dermatology, Erasmus MC, Rotterdam, The Netherlands. This work was supported by a grant from the Netherlands Organisation for Health Research and Development (project number 113303005) (to WEV) and from the Sherman Foundation (to WEV). We thank Linda Broer, PhD, for the creation of imputed data, with the support of Marijn Verkerk and Carolina Medina-Gomez, MSc, for the analysis setup. We would like to thank Karol Estrada PhD, Fernando Rivadeneira PhD, and Anis Abuseiris (Erasmus MC Rotterdam, The Netherlands), for their help in creating GRIMP, and we thank BigGRID for access to their grid computing resources.

SardiNIA
We thank all the volunteers who generously participated in this study and made this research possible. This research was supported by National Human Genome Research Institute grants HG005581, HG005552, HG006513, HG007022 and HG007089; by National Heart, Lung, and Blood Institute grant HL117626; by the Intramural Research Program of the US National Institutes of Health, National Institute on Aging, contracts N01-AG-1-2109 and HHSN271201100005C; by Sardinian Autonomous Region (L.R. 7/2009) grant cRP3-154; by the PB05 InterOmics MIUR Flagship Project; by grant FaReBio2011 'Farmaci e Reti Biotecnologiche di Qualità'. The study including the Informed Consent for participants was reviewed and approved by institutional review boards for the Istituto di Neurogenetica e Neurofarmacologia (INN; Cagliari, Italy), for the MedStar Research Institute (responsible for intramural research at the National Institutes of Aging, Baltimore, Maryland, United States) and for the University of Michigan (Ann Arbor, Michigan, United States).

SHIP-START/SHIP-TREND
SHIP is part of the Community Medicine Research Net of the University Medicine Greifswald, Germany, which is supported by the Federal Ministry of Education and Research (grants no. 01ZZ9603, 01ZZ0103, and 01ZZ0403), the Ministry of Cultural Affairs as well as the Social Ministry of the Federal State of Mecklenburg-West Pomerania, and the network 'Greifswald Approach to Individualized Medicine (GANI_MED)' funded by the Federal Ministry of Education and Research (grant 03IS2061A). Genome-wide data have been supported by the Federal Ministry of Education and Research (grant no. 03ZIK012) and a joint grant from Siemens Healthineers, Erlangen, Germany and the Federal State of Mecklenburg- West Pomerania. The University of Greifswald is a member of the Caché Campus program of the InterSystems GmbH. The medical ethics committee of the University of Greifswald approved the study protocol, and oral and written informed consents were obtained from each of the study participants.

SIBLOS
Kaatje Toye: Study Coordinator SIBLOS, Ghent University. The study protocol was approved by the ethical committee of the Ghent University Hospital (project 2001/60) and written informed consent was obtained from all participants.

TwinsUK
TwinsUK is funded by the Wellcome Trust, Medical Research Council, European Union, the National Institute for Health Research (NIHR)-funded BioResource, Clinical Research Facility and Biomedical Research Centre based at Guy's and St Thomas' NHS Foundation Trust in partnership with King's College London. Ethical approval was granted by the National Research Ethics Service London-Westminster, the St Thomas' Hospital Research Ethics Committee (EC04/015 and 07/H0802/84).

UK Biobank
Ethics approval for the UK Biobank study was obtained from the North West Centre for Research Ethics Committee (11/NW/0382).

Val Borbera
We thank all the participants in the project, the San Raffaele Hospital MDs who contributed to clinical data collection, Prof. Daniela Toniolo and Prof. Clara Camaschella who coordinated the data collection, Corrado Masciullo and Massimiliano Cocca for the database informatics. The research was supported by funds from Compagnia di San Paolo, Torino, Italy; Fondazione Cariplo, Italy; Telethon Italy; Ministry of Health, Ricerca Finalizzata 2008 and 2011-2012 and Public Health Genomics Project 2010. The study was reviewed and approved by the Ethics Committee of the "San Raffaele" Hospital and of the Piemonte Region.

Women's Genome Health Study (WGHS)
The WGHS is supported by the National Heart, Lung, and Blood Institute (HL043851 and HL080467) and the National Cancer Institute (CA047988 and UM1CA182913), with additional funding for genotyping provided by Amgen. Additional funding was also provided to Dr. Mora by an investigator-initiated grant from Atherotech Diagnostics (for the thyroid measurements) and from the National Heart, Lung, and Blood Institute (K24 HL136852). Analysis in the WGHS is conducted with consent of participants and local IRB approval. Christina Ellervik is partly funded by the Laboratory Medicine Endowment Fund of Boston Children's Hospital.

Supplementary Figure 1: Overview of sample sizes per cohort per thyroid function trait

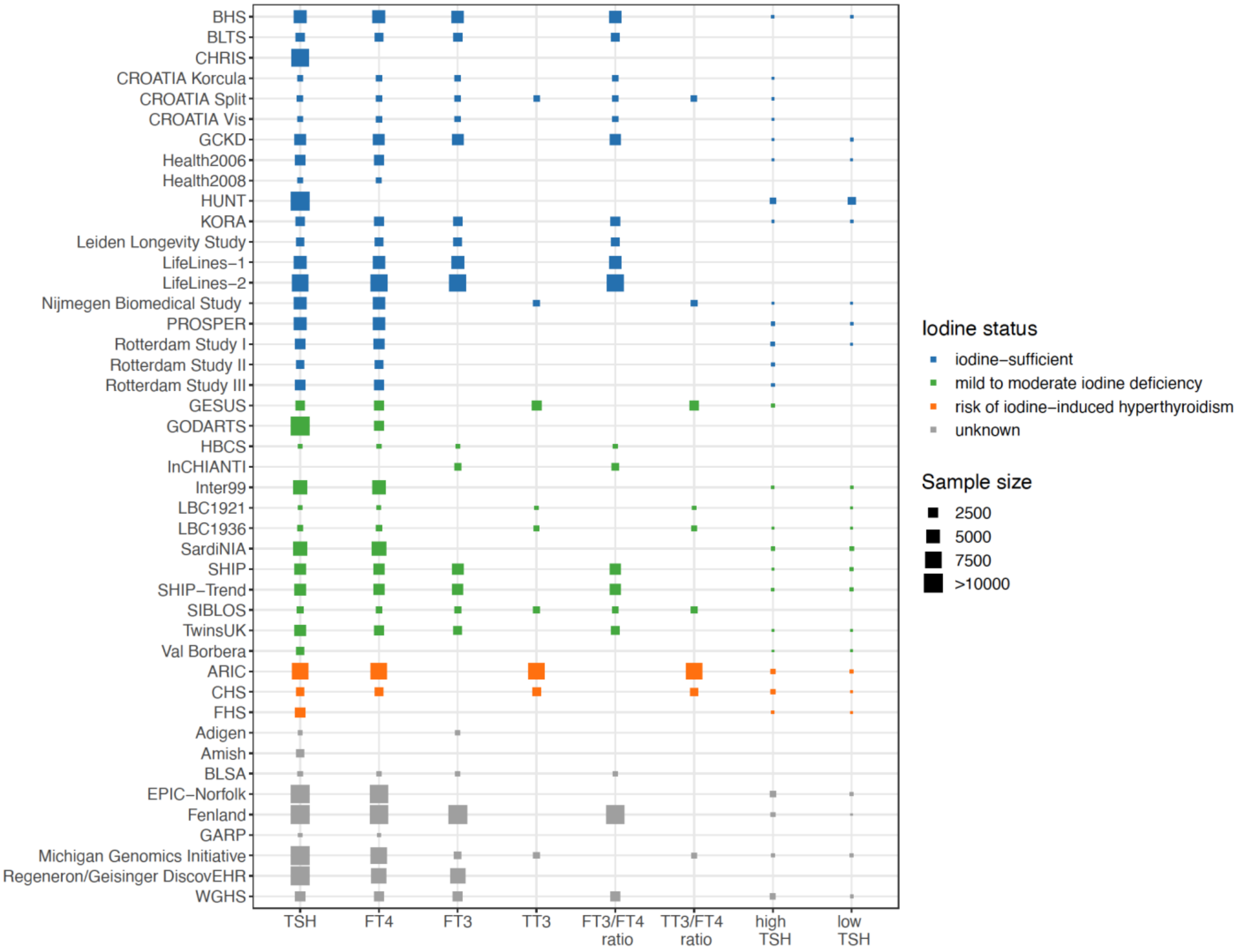

Overview of the sample sizes included in this GWAS meta-analysis for the respective traits per cohort including information of the iodine status in that specific cohort. The size of the squares indicates the size of the sample. The colors refer to the specific iodine status of the included cohort.

Supplementary Figure 2: Mirrored Manhattan plot FT3 and TT3

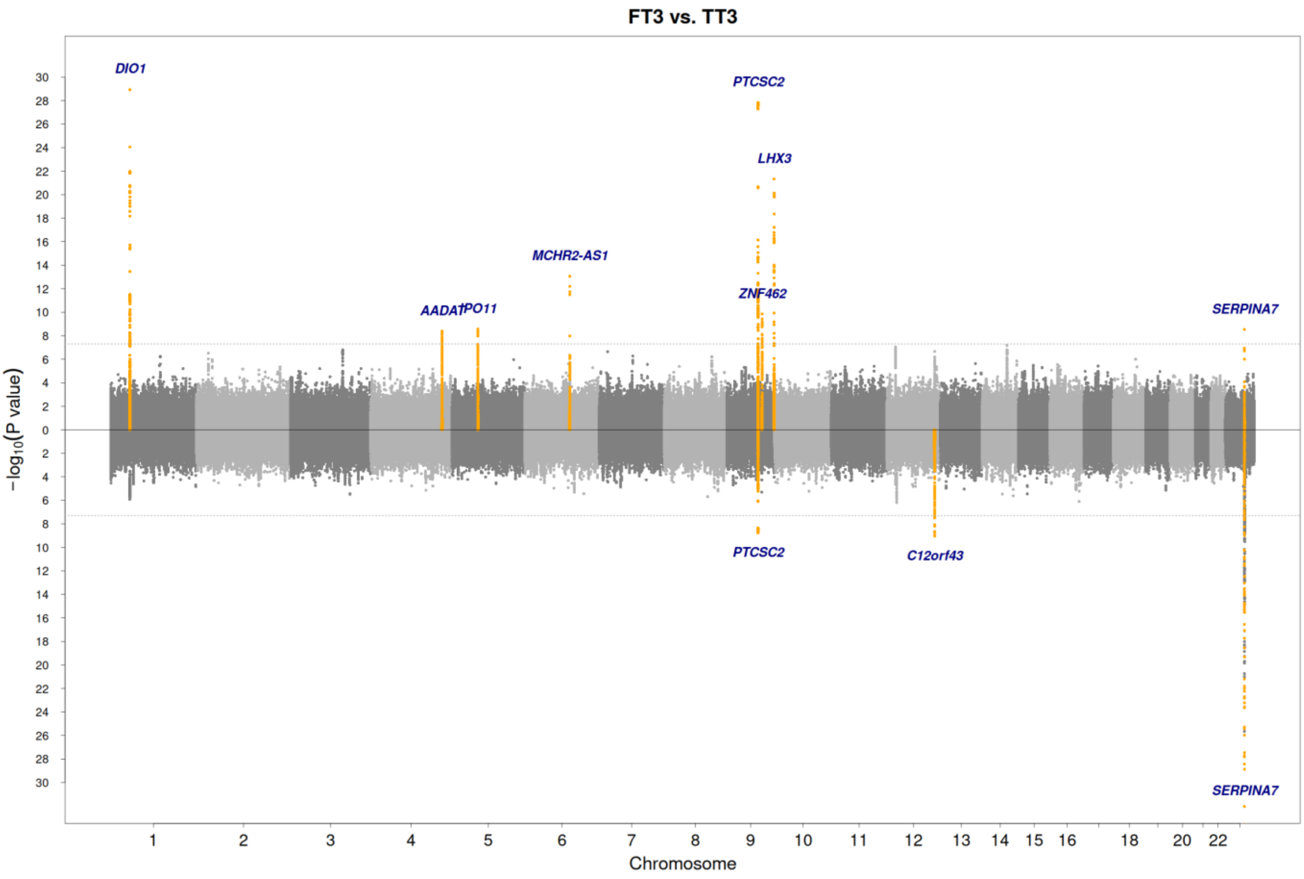

Manhattan plot of the GWAS meta-analysis results for FT3 (n = 59,061, upper panel) and TT3 (n = 15,829, lower panel) contrasted with each other. Variants are plotted on the x-axis according to their position on each chromosome with the -log10(p-value) of the association test (2-sided, z-statistics) on the y-axis. The horizontal line indicates the threshold for genome-wide significance, p = $5x10^{-8}$. Previously not known associated loci are colored in orange.

Supplementary Figure 3: Mirrored Manhattan plot FT3/FT4 ratio and TT3/FT4 ratio

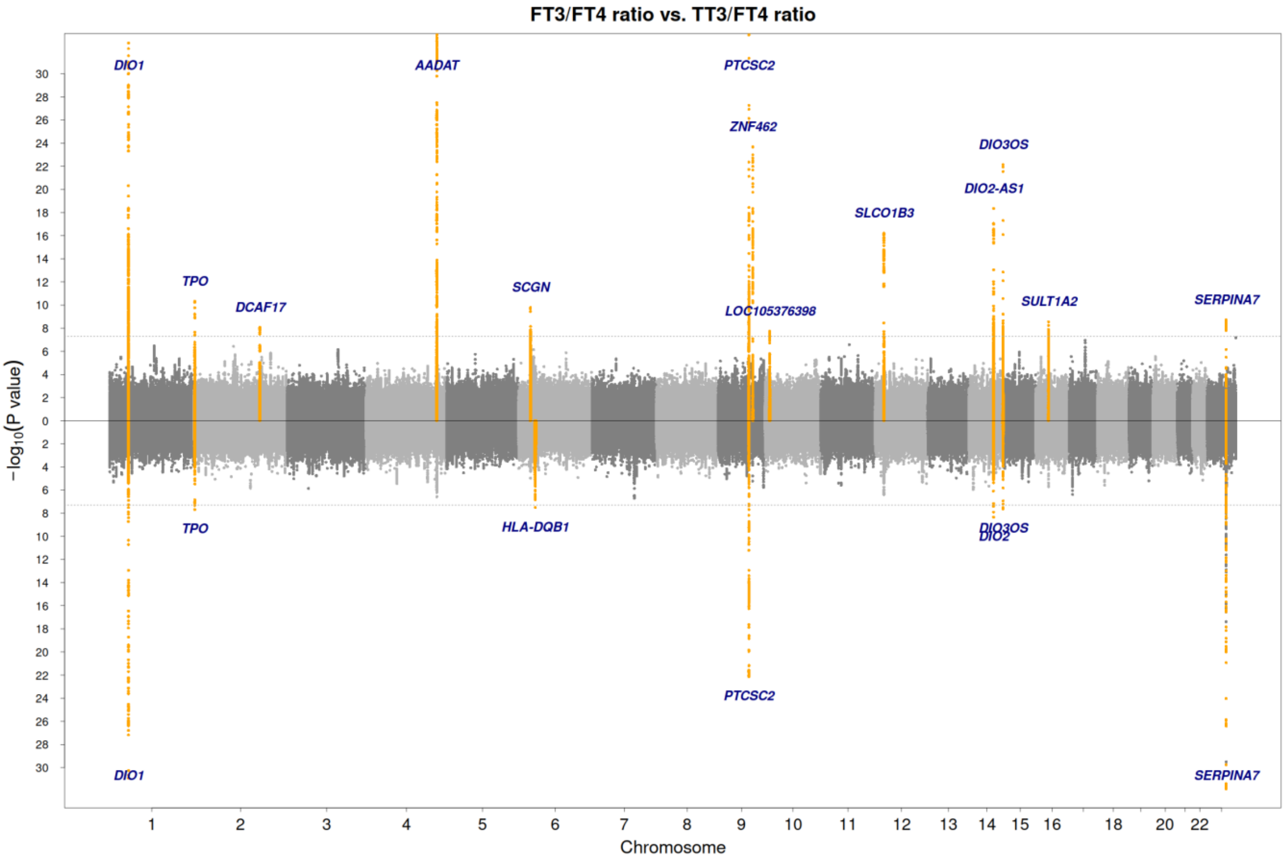

Manhattan plot of the GWAS meta-analysis results for FT3/FT4 ratio (n = 51,095, upper panel) and TT3/FT4 ratio (n = 15,510, lower panel) contrasted with each other. Variants are plotted on the x-axis according to their position on each chromosome with the -log10(p-value) of the association test (2-sided, z-statistics) on the y-axis. The horizontal line indicates the threshold for genome-wide significance, $p = 5x10^{-8}$. Previously not known associated loci are colored in orange.

Supplementary Figure 4: Mirrored Manhattan plot high TSH and low TSH

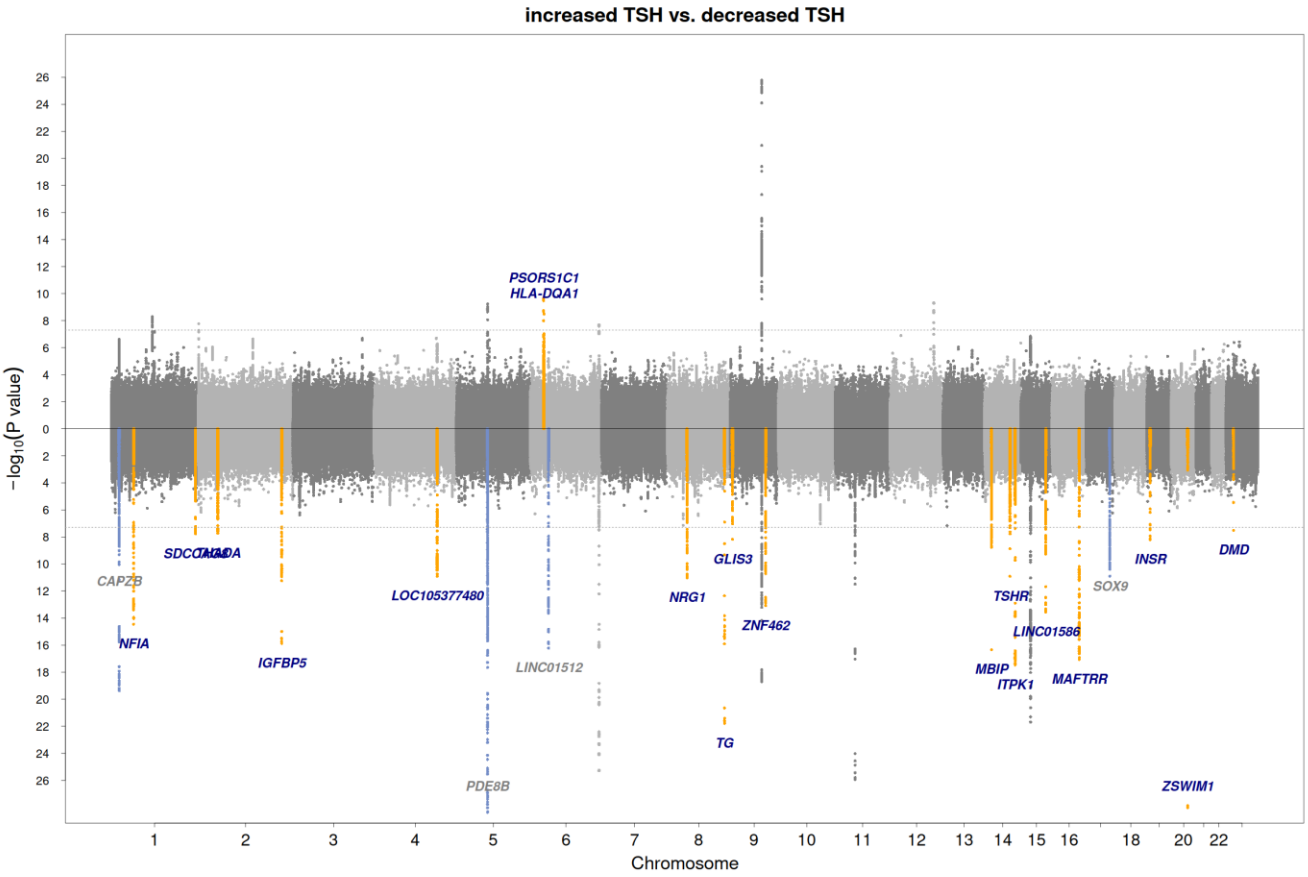

Manhattan plot of the GWAS meta-analysis results for high (n = 153,241, upper panel) and low TSH (n = 141,549, lower panel) contrasted with each other. Variants are plotted on the x-axis according to their position on each chromosome with the -log10(p-value) of the association test (2-sided, z-statistics) on the y-axis. The horizontal line indicates the threshold for genome-wide significance, p = $5x10^{-8}$. Previously not known associated loci are colored in orange, and novel independent associations within known loci are colored in light blue.

Supplementary Figure 5: Beta-beta plots of TSH and FT4 index variants versus previous GWAS

a

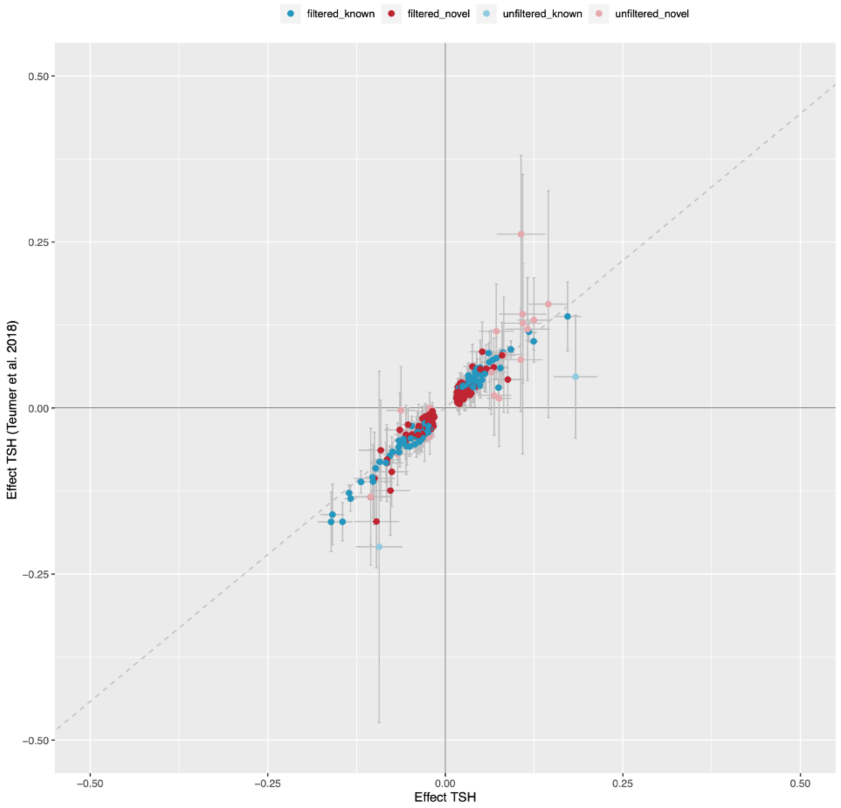

b

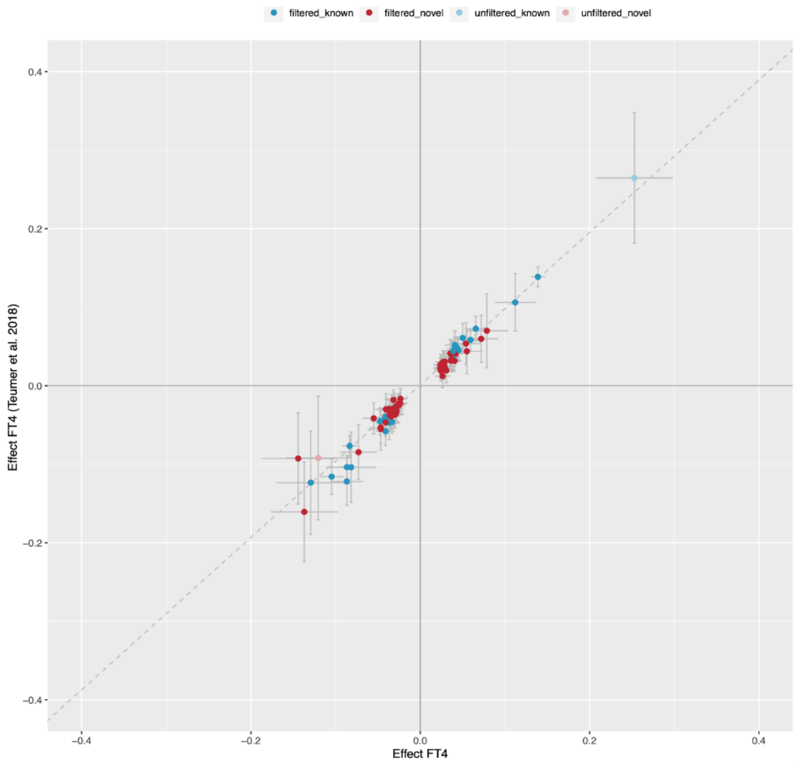

Betas of the significant independent variants for **a)** TSH (n=258) and **b)** FT4 (n=85) were compared with the previous reference range TSH and FT4 GWAS (Teumer *et al.* 2018). Variants were considered as known (light/dark blue) if they correlate (R^2>0.1) with a previously known variant (see Methods). Novel variants are depicted in light/dark red. If a variant was not present in the published sample size and minor allele frequency filtered dataset (dark shade), unfiltered results (light shade) were used. The bars represent the 95% confidence intervals of the betas. For one TSH association (rs375911171), no results in the summary statistics of Teumer *et al.* were available.

Supplementary Figure 6: Quantile-quantile plots for all thyroid function traits

a) TSH

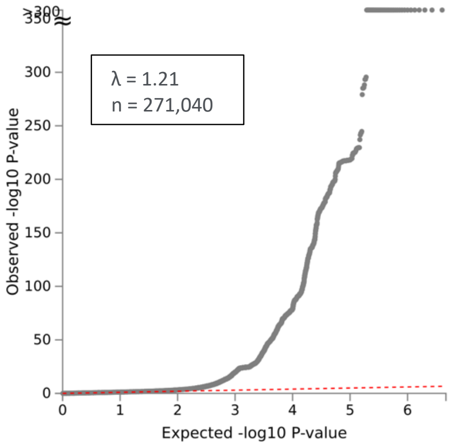

b) FT4

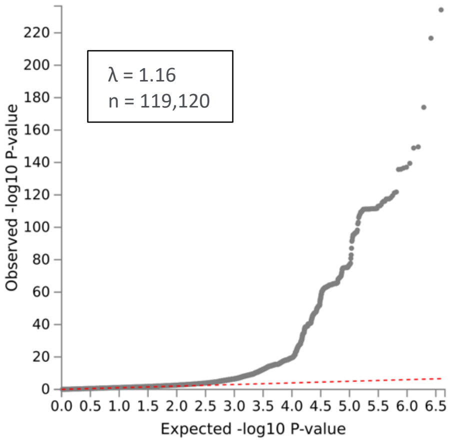

c) FT3

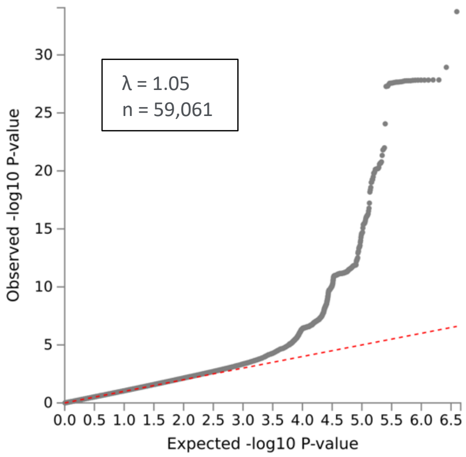

d) TT3

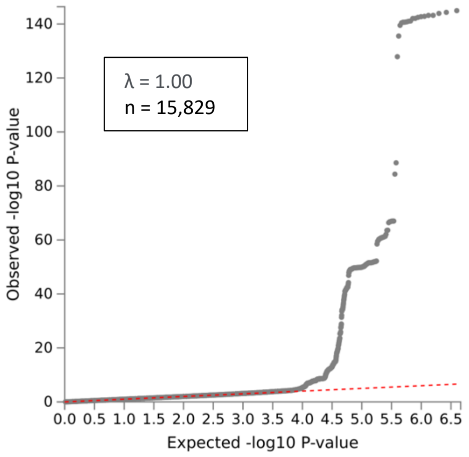

e) FT3/FT4 ratio

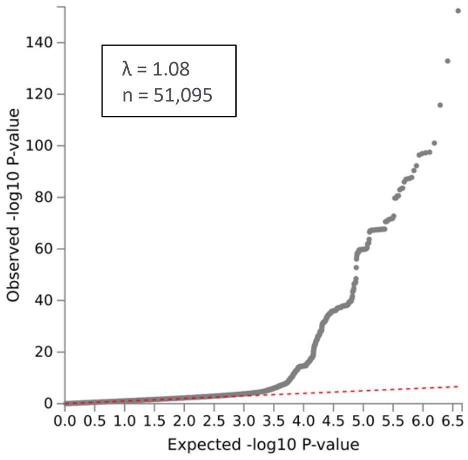

f) TT3/FT4 ratio

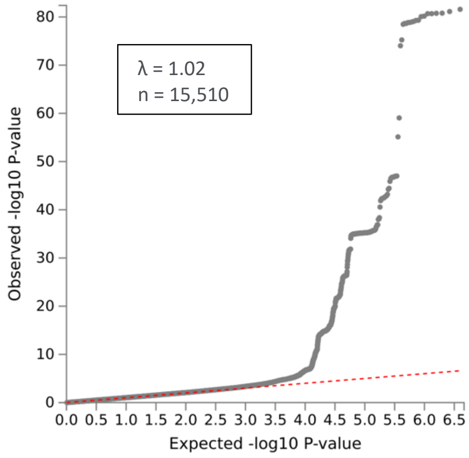

g) high TSH

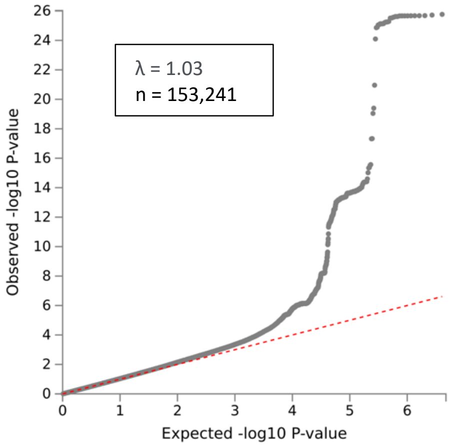

h) low TSH

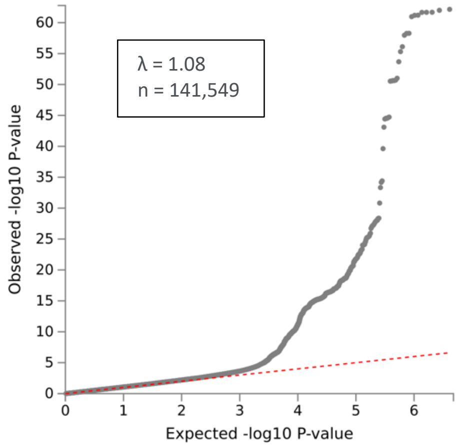

Quantile-quantile (QQ) plots of the GWAS meta-analysis results for **a)** TSH, **b)** FT4, **c)** FT3, **d)** TT3, **e)** FT3/FT4 ratio, **f)** TT3/FT4 ratio, **g)** high TSH and **h)** low TSH in the overall sample. On the y-axis, the observed p-values of the association test are plotted against the expected distribution under the null hypothesis of no association on the x-axis.
λ: lambda, genomic control parameter; n: sample size.

Supplementary Figure 7: Fine-mapping results

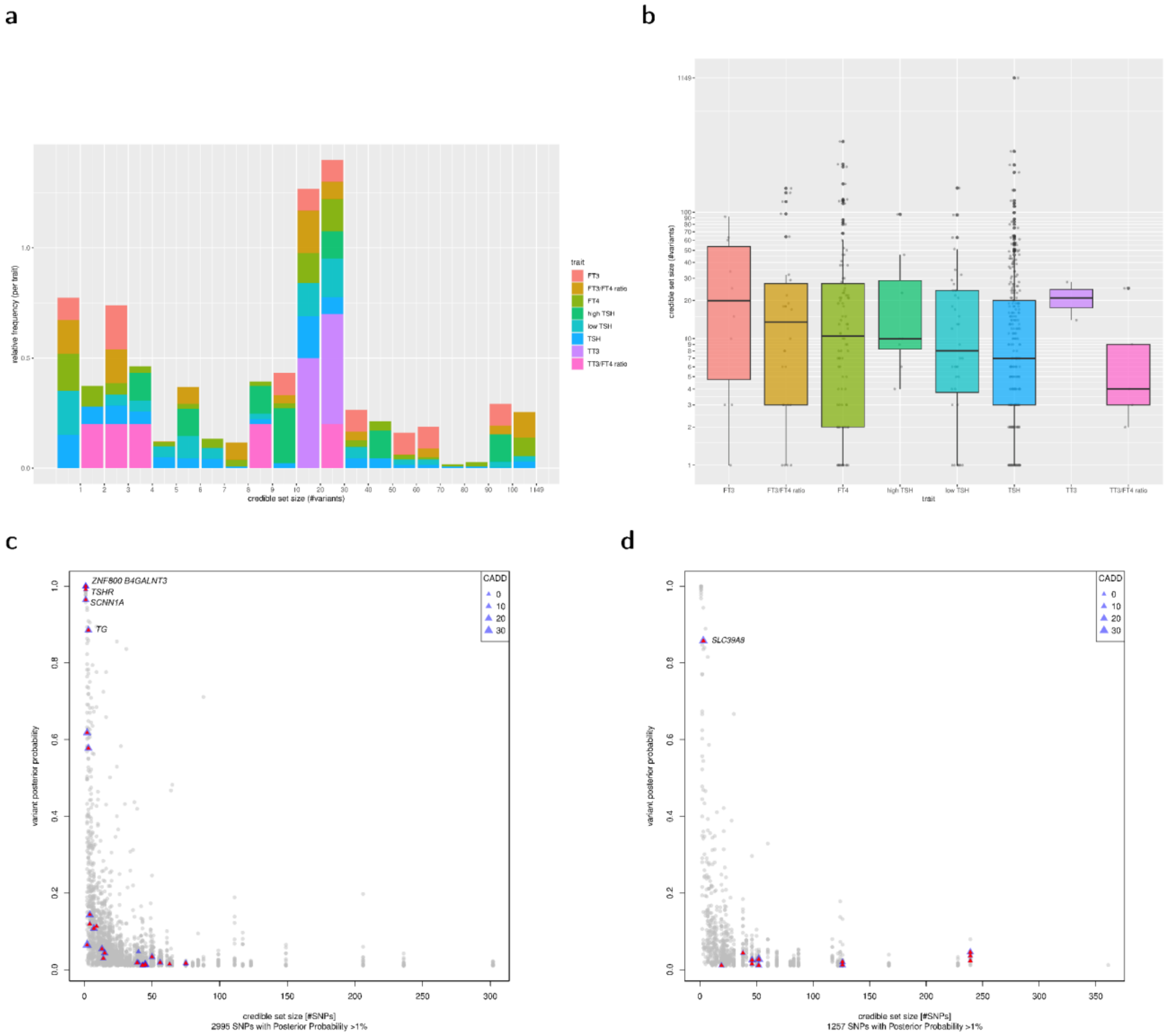

Fine-mapping summary and functional annotation of potentially causal variants.
Panel **a** provides a histogram of the credible set size (relative to the number of total significant loci per trait) per trait, panel **b** shows the boxplots of the number of variants of a credible set per trait. In the boxplots, the black horizontal line represents the median, the lower and upper hinges correspond to the 25th and 75th percentiles, the upper and lower whisker extend from the hinges to the 1.5 * inter-quartile range, where values outside this range are plotted separately in black.
In panels **c** (TSH) and **d** (FT4), exonic variants are indicated by filled red triangles, and missense variants are marked by blue triangles, with size proportional to the variant CADD score. The labels show the gene, and are restricted to variants with high posterior probability (≥0.8) of driving the association signal.

Supplementary Figure 8. Colocalizations of high and low TSH in the tissues of the HTP axis

a

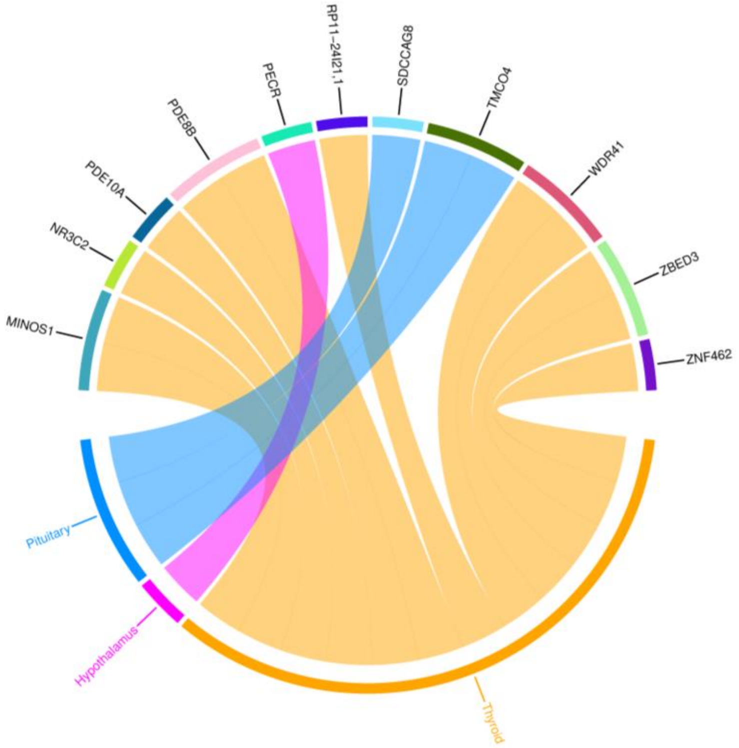

b

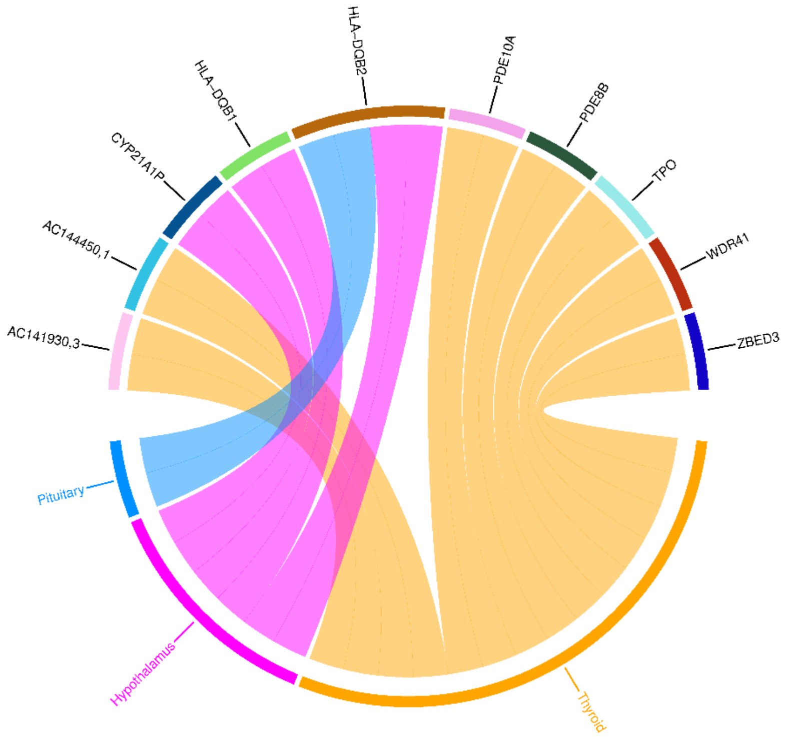

The plots show the colocalizations of low TSH (**a**) and high TSH (**b**) in the tissues of the hypothalamus-pituitary-thyroid (HPT) axis.

Supplementary Figure 9. Top 10 canonical pathways for TSH, FT4, FT3/FT4 ratio and low TSH.

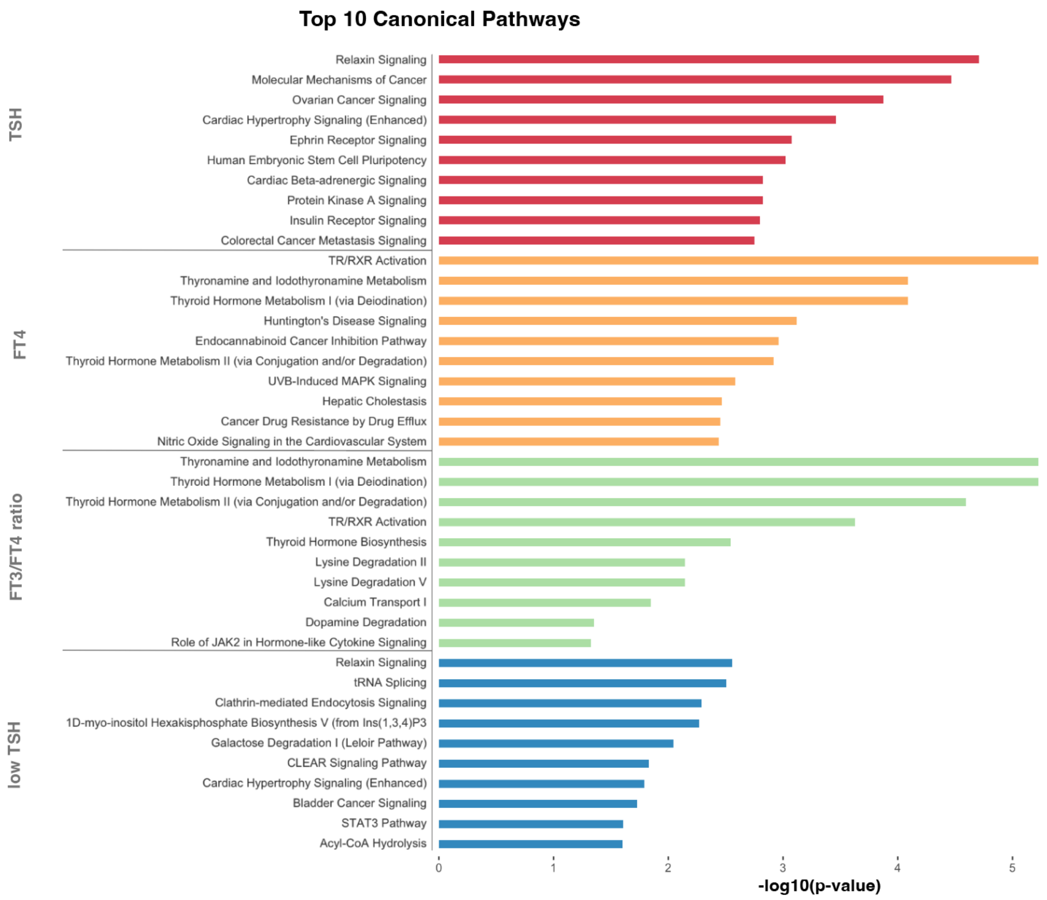

The top ten significantly associated pathways for TSH, FT4, FT3/FT4 ratio and low TSH are shown (y-axis) with the corresponding -log^{10}(p-value) (x-axis) using IPA analyses. P-values were calculated using the right-tailed fisher exact test and corrected for multiple testing using the Benjamini-Hochberg correction.

Supplementary Figure 10: Tissue expression analysis using MAGMA

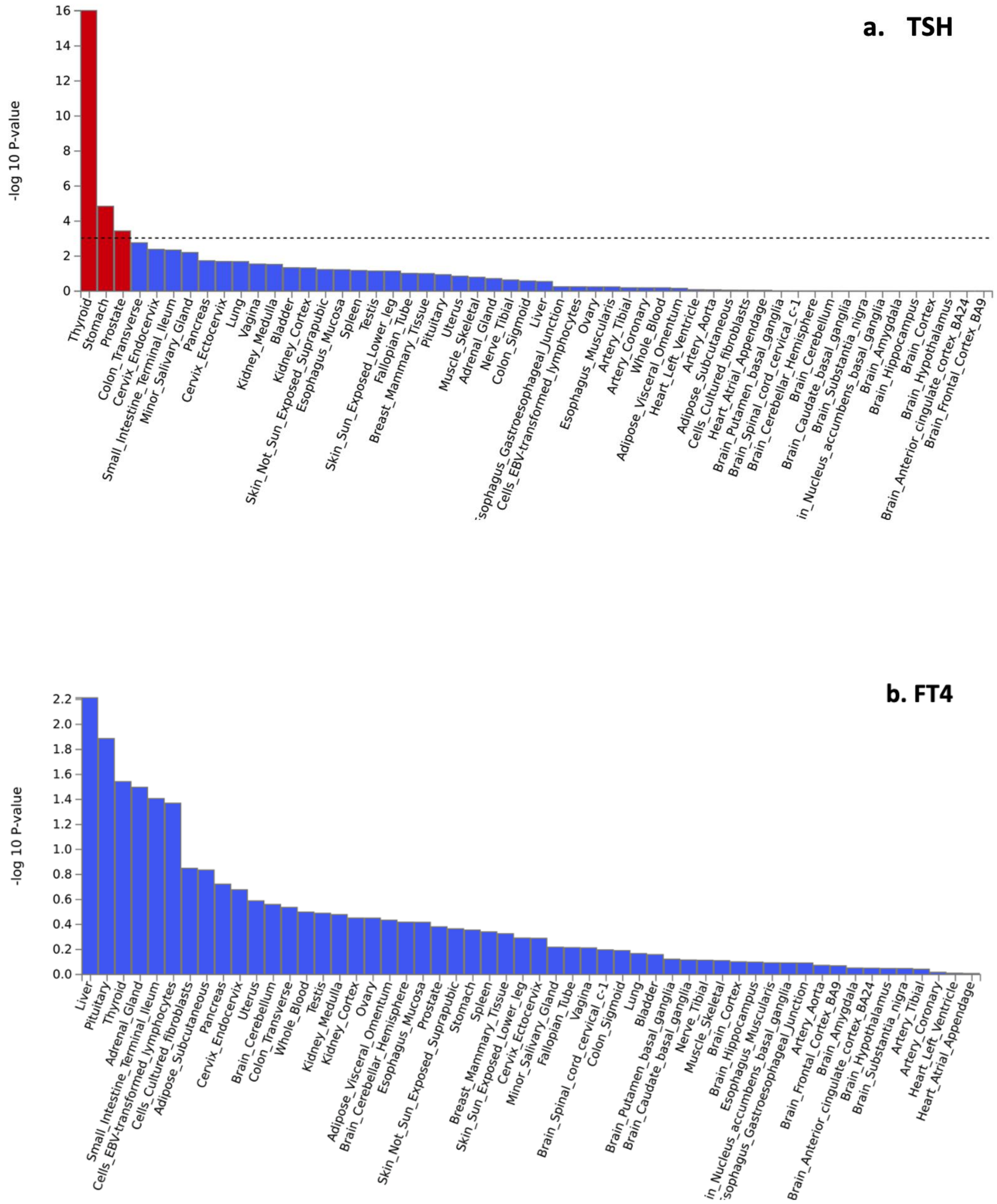

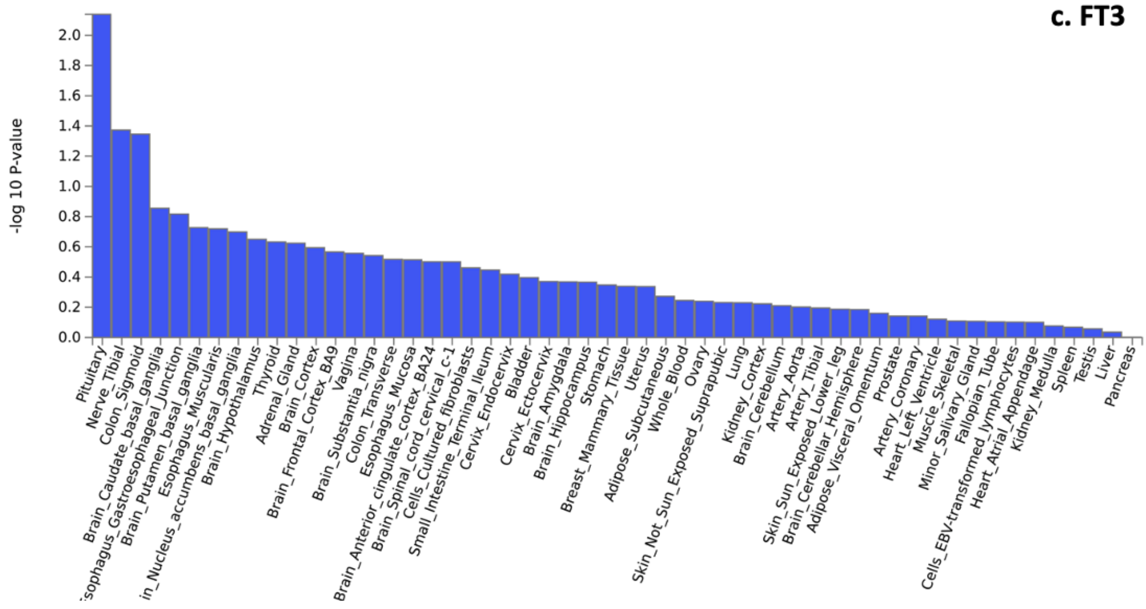
c. FT3
-log 10 P-value
2.0
1.8
1.6
1.4
1.2
1.0
0.8
0.6
0.4
0.2
0.0
Pituitary
Nerve_Tibial
Colon_Sigmoid
Brain_Caudate_basal_ganglia
sophagus_Gastroesophageal_Junction
Brain_Putamen_basal_ganglia
Esophagus_Muscularis
in_Nucleus_accumbens_basal_ganglia
Brain_Hypothalamus
Thyroid
Adrenal_Gland
Brain_Cortex
Brain_Frontal_Cortex_BA9
Vagina
Brain_Substantia_nigra
Colon_Transverse
Esophagus_Mucosa
Brain_Anterior_cingulate_cortex_BA24
Brain_Spinal_cord_cervical_c-1
Cells_Cultured_fibroblasts
Small_Intestine_Terminal_Ileum
Cervix_Endocervix
Bladder
Cervix_Ectocervix
Brain_Amygdala
Brain_Hippocampus
Stomach
Breast_Mammary_Tissue
Uterus
Adipose_Subcutaneous
Whole_Blood
Ovary
Skin_Not_Sun_Exposed_Suprapubic
Lung
Kidney_Cortex
Brain_Cerebellum
Artery_Aorta
Artery_Tibial
Skin_Sun_Exposed_Lower_leg
Brain_Cerebellar_Hemisphere
Adipose_Visceral_Omentum
Prostate
Artery_Coronary
Heart_Left_Ventricle
Muscle_Skeletal
Minor_Salivary_Gland
Fallopian_Tube
Cells_EBV-transformed_lymphocytes
Heart_Atrial_Appendage
Kidney_Medulla
Spleen
Testis
Liver
Pancreas

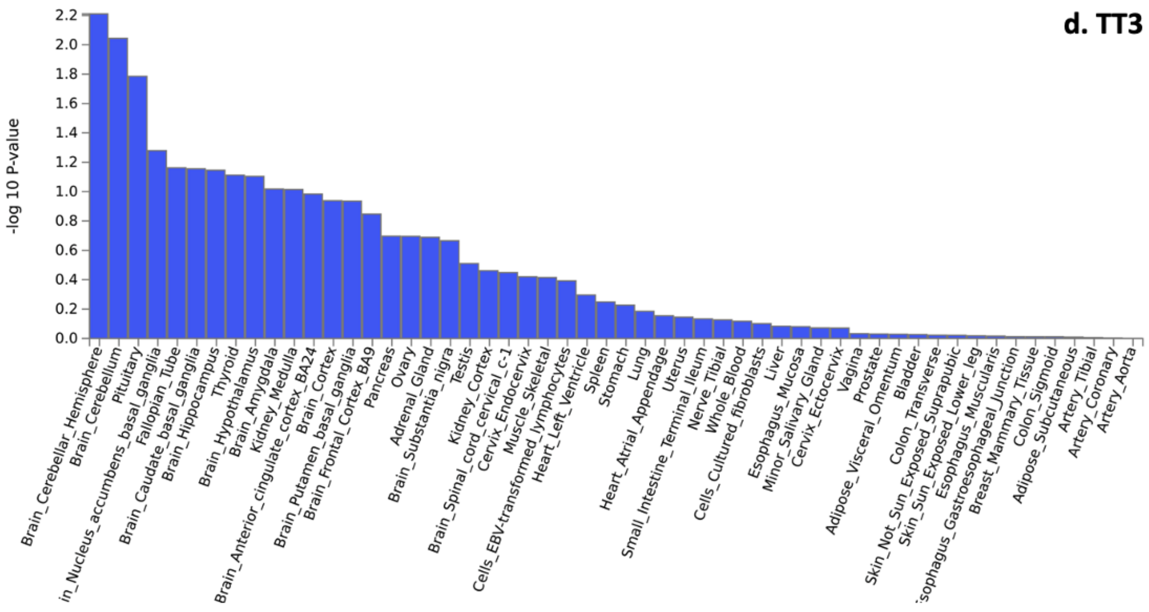
d. TT3
-log 10 P-value
2.2
2.0
1.8
1.6
1.4
1.2
1.0
0.8
0.6
0.4
0.2
0.0
Brain_Cerebellar_Hemisphere
Brain_Cerebellum
Pituitary
in_Nucleus_accumbens_basal_ganglia
Fallopian_Tube
Brain_Caudate_basal_ganglia
Brain_Hippocampus
Thyroid
Brain_Hypothalamus
Brain_Amygdala
Kidney_Medulla
Brain_Anterior_cingulate_cortex_BA24
Brain_Cortex
Brain_Putamen_basal_ganglia
Brain_Frontal_Cortex_BA9
Pancreas
Ovary
Adrenal_Gland
Brain_Substantia_nigra
Testis
Kidney_Cortex
Brain_Spinal_cord_cervical_c-1
Cervix_Endocervix
Muscle_Skeletal
Cells_EBV-transformed_lymphocytes
Heart_Left_Ventricle
Spleen
Stomach
Lung
Heart_Atrial_Appendage
Uterus
Small_Intestine_Terminal_Ileum
Nerve_Tibial
Whole_Blood
Cells_Cultured_fibroblasts
Liver
Esophagus_Mucosa
Minor_Salivary_Gland
Cervix_Ectocervix
Vagina
Prostate
Adipose_Visceral_Omentum
Bladder
Colon_Transverse
Skin_Not_Sun_Exposed_Suprapubic
Skin_Sun_Exposed_Lower_leg
Esophagus_Muscularis
sophagus_Gastroesophageal_Junction
Breast_Mammary_Tissue
Colon_Sigmoid
Adipose_Subcutaneous
Artery_Tibial
Artery_Coronary
Artery_Aorta

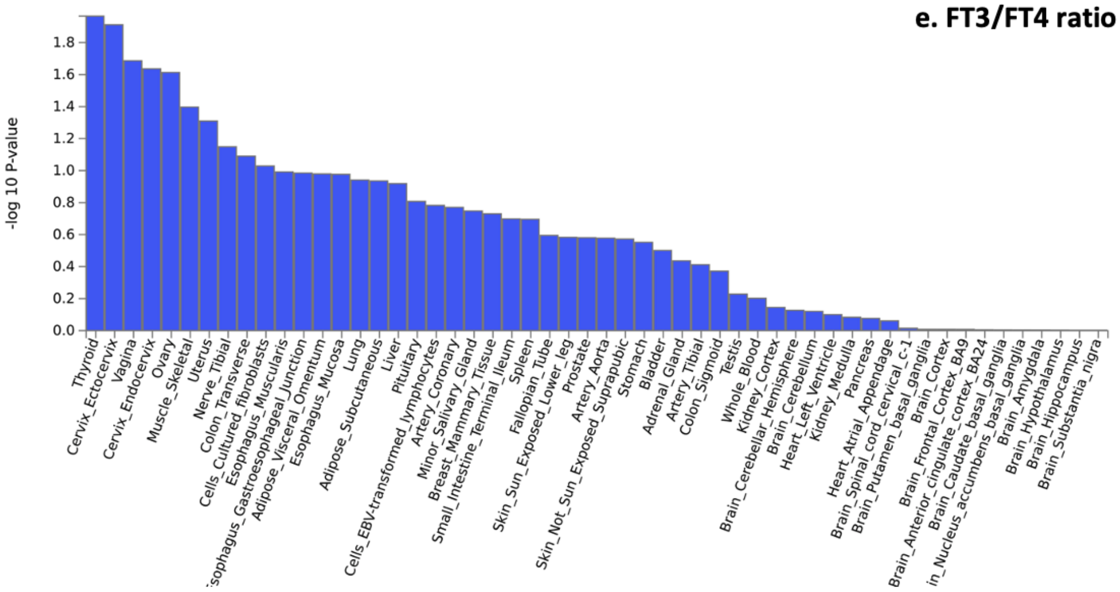
e. FT3/FT4 ratio
-log 10 P-value
0.0
0.2
0.4
0.6
0.8
1.0
1.2
1.4
1.6
1.8
Thyroid
Cervix_Ectocervix
Vagina
Cervix_Endocervix
Ovary
Muscle_Skeletal
Uterus
Nerve_Tibial
Colon_Transverse
Cells_Cultured_fibroblasts
Esophagus_Muscularis
sophagus_Gastroesophageal_Junction
Adipose_Visceral_Omentum
Esophagus_Mucosa
Lung
Adipose_Subcutaneous
Liver
Pituitary
Cells_EBV-transformed_lymphocytes
Artery_Coronary
Minor_Salivary_Gland
Breast_Mammary_Tissue
Small_Intestine_Terminal_Ileum
Spleen
Fallopian_Tube
Skin_Sun_Exposed_Lower_leg
Prostate
Artery_Aorta
Skin_Not_Sun_Exposed_Suprapubic
Stomach
Bladder
Adrenal_Gland
Artery_Tibial
Colon_Sigmoid
Testis
Whole_Blood
Kidney_Cortex
Brain_Cerebellar_Hemisphere
Brain_Cerebellum
Heart_Left_Ventricle
Kidney_Medulla
Pancreas
Heart_Atrial_Appendage
Brain_Spinal_cord_cervical_c-1
Brain_Putamen_basal_ganglia
Brain_Cortex
Brain_Frontal_Cortex_BA9
Brain_Anterior_cingulate_cortex_BA24
Brain_Caudate_basal_ganglia
in_Nucleus_accumbens_basal_ganglia
Brain_Amygdala
Brain_Hypothalamus
Brain_Hippocampus
Brain_Substantia_nigra

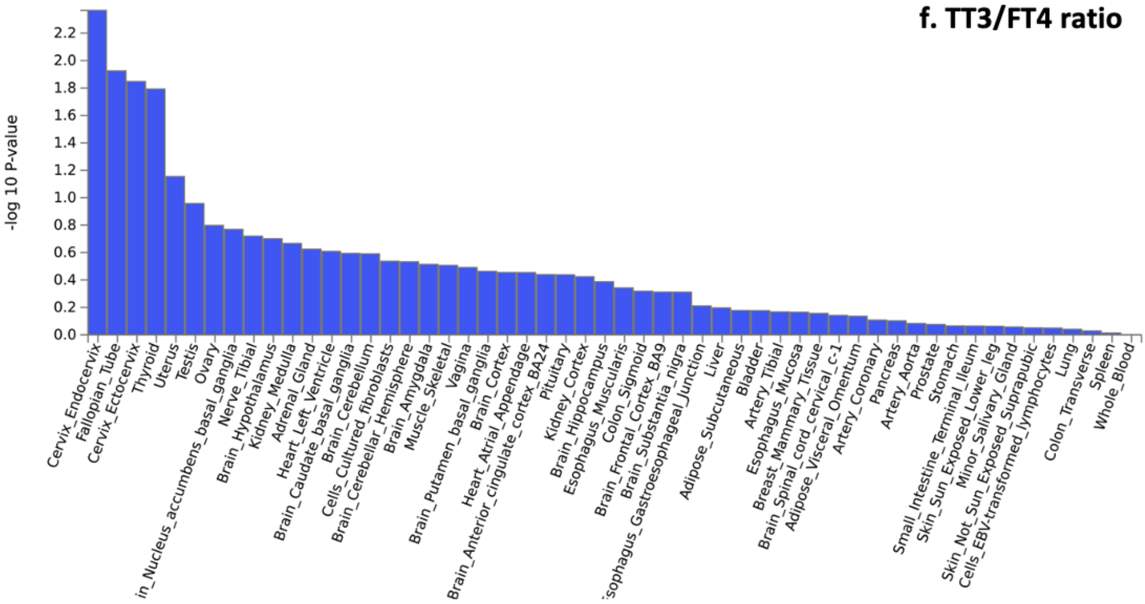
f. TT3/FT4 ratio
-log 10 P-value
0.0
0.2
0.4
0.6
0.8
1.0
1.2
1.4
1.6
1.8
2.0
2.2
Cervix_Endocervix
Fallopian_Tube
Cervix_Ectocervix
Thyroid
Uterus
Testis
Ovary
in_Nucleus_accumbens_basal_ganglia
Nerve_Tibial
Brain_Hypothalamus
Kidney_Medulla
Adrenal_Gland
Heart_Left_Ventricle
Brain_Caudate_basal_ganglia
Brain_Cerebellum
Cells_Cultured_fibroblasts
Brain_Cerebellar_Hemisphere
Brain_Amygdala
Muscle_Skeletal
Vagina
Brain_Putamen_basal_ganglia
Brain_Cortex
Heart_Atrial_Appendage
Brain_Anterior_cingulate_cortex_BA24
Pituitary
Kidney_Cortex
Brain_Hippocampus
Esophagus_Muscularis
Colon_Sigmoid
Brain_Frontal_Cortex_BA9
Brain_Substantia_nigra
sophagus_Gastroesophageal_Junction
Liver
Adipose_Subcutaneous
Bladder
Artery_Tibial
Esophagus_Mucosa
Breast_Mammary_Tissue
Brain_Spinal_cord_cervical_c-1
Adipose_Visceral_Omentum
Artery_Coronary
Pancreas
Artery_Aorta
Prostate
Stomach
Small_Intestine_Terminal_Ileum
Skin_Sun_Exposed_Lower_leg
Minor_Salivary_Gland
Skin_Not_Sun_Exposed_Suprapubic
Cells_EBV-transformed_lymphocytes
Lung
Colon_Transverse
Spleen
Whole_Blood

g. High TSH

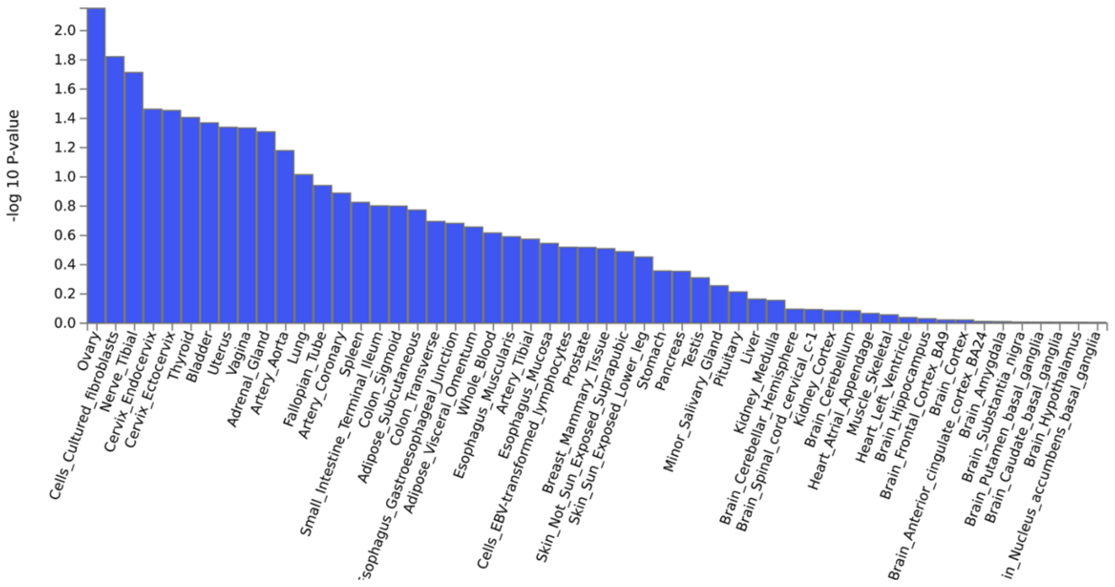

h. Low TSH

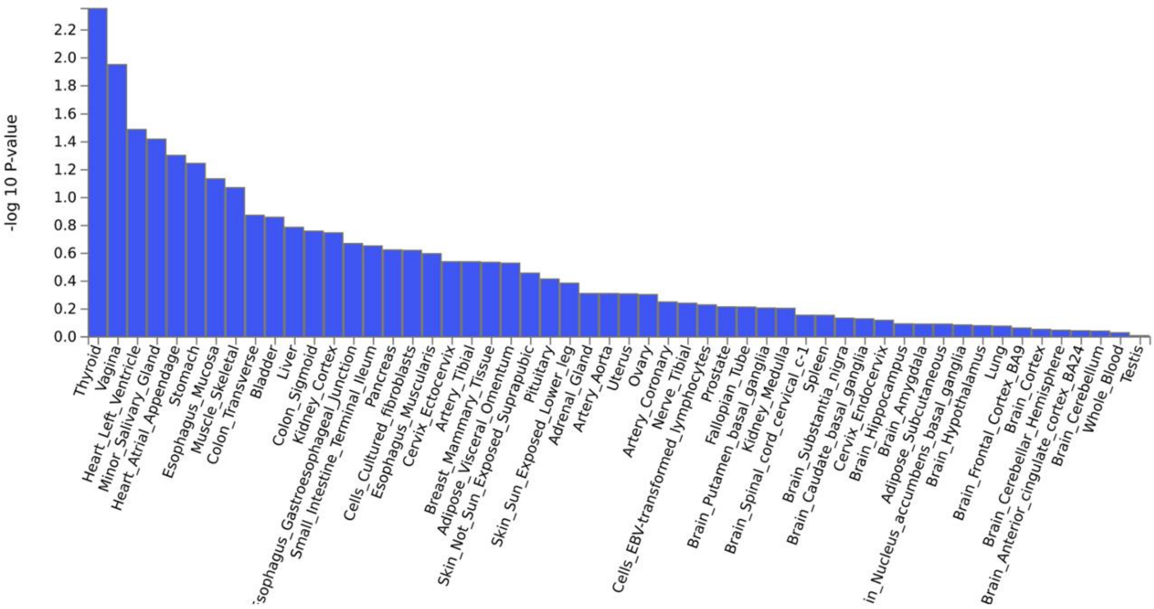

Overview of the results of the MAGMA tissue enrichment analysis (FUMA) for the thyroid function parameters using GTEx data for 54 tissue types. Nominal $-\log_{10}$ p-values obtained from the MAGMA gene-property test are shown on the y-axis, the respective tested tissues on the x-axis, and the dotted line shows the significance level after Bonferroni correction for the number of tissues tested ($p = 9.3x10^{-4}$). Red bars show significant tissue enrichment results, blue bars non-significant results. Instead of $r^2 = 0.01$, $r^2 = 0.05$ was used to identify independent variants (lowest option in FUMA). All other parameters used in the main meta-analysis of this GWAS were used as input parameter in FUMA.

Supplementary Figure 11. Risk of thyroid cancer in Icelandic individuals by various thyroid function parameter polygenic risk scores.

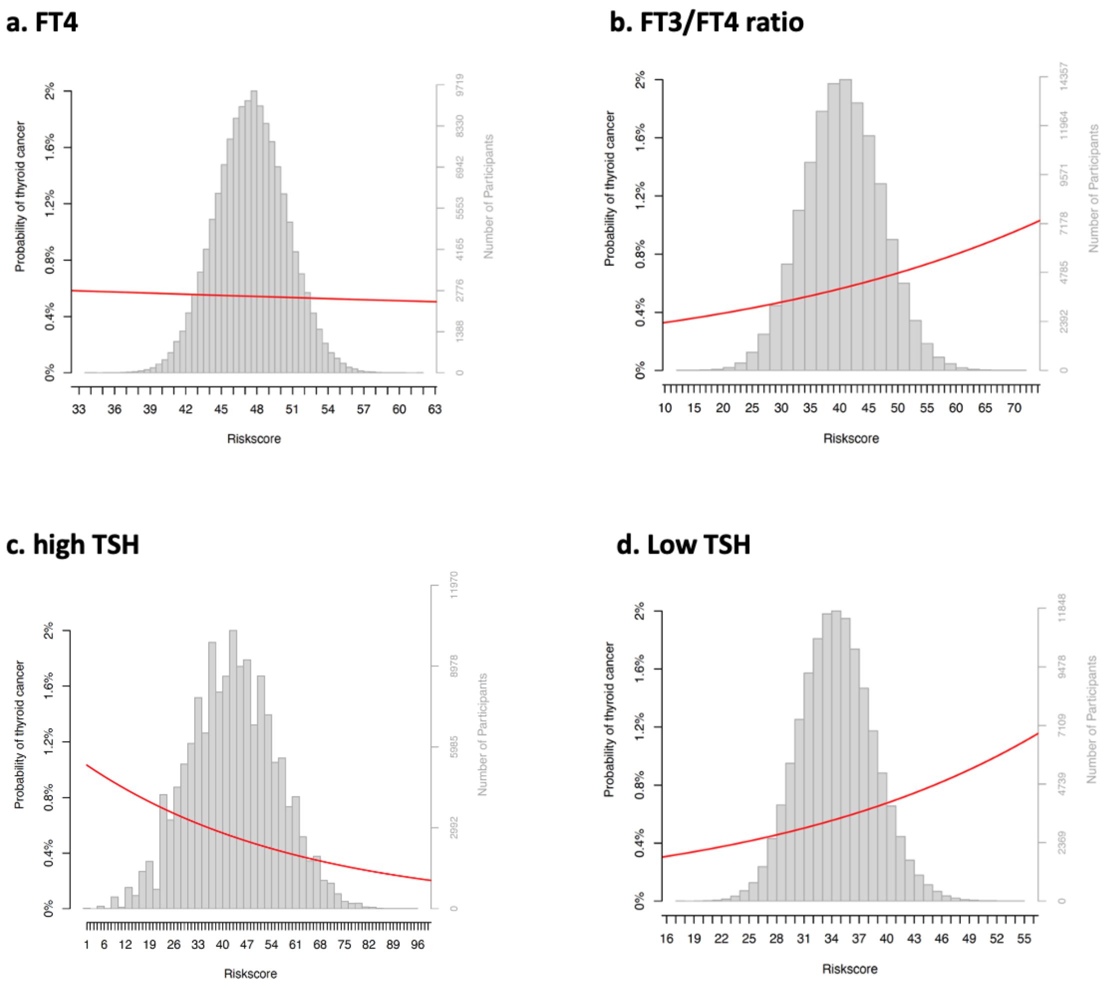

Plots of the associations of genetic risk scores with thyroid cancer in deCODE (**a;** N_{case}=754, $N_{control}$=138089, $N_{control}$=121394, **b;** N_{case}=682, $N_{control}$=118963, **c**; N_{case}=792, $N_{control}$=148843, **d;** N_{case}=676, $N_{control}$=117809). The y-axis shows the probability of thyroid cancer including the p-value of the association test of the trait on the risk score. The x-axis shows the percentage of risk alleles carried based on a weighted polygenic score. The histogram shows the distribution of the GRS in the study sample per trait. N_{case}: sample size of cases. $N_{control}$: sample size of controls.

Supplementary Figure 12. Mendelian Randomization forest plot TSH vs thyroid cancer

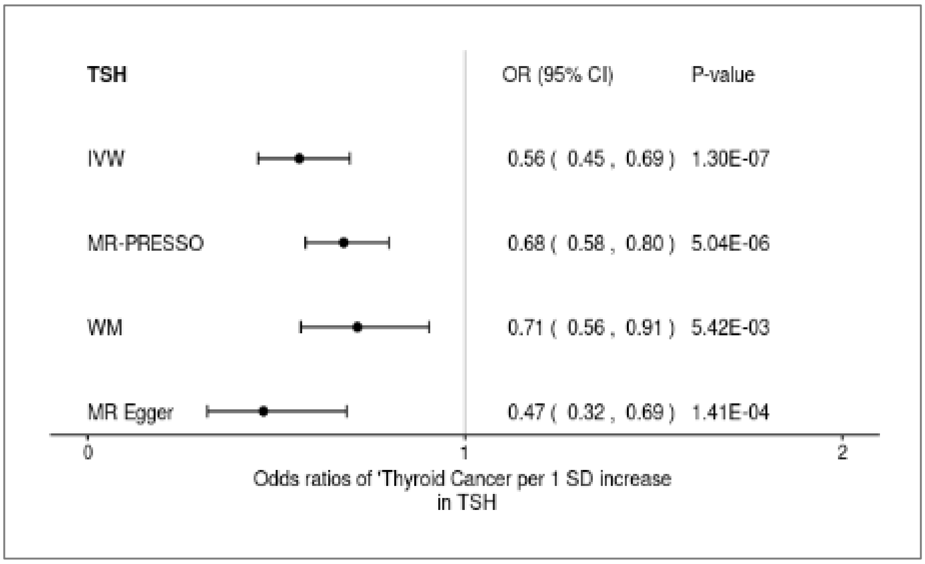

Odds ratios (95% confidence intervals) and p-values of the different MR analyses on reference range TSH (n = 271,040) and thyroid cancer (n = 3,100 cases and 287,550 controls). MR: Mendelian randomization, IVW: inverse variance weighted MR, WM: weighted median MR. MR PRESSO: Mendelian randomization pleiotropy residual sum and outlier test to identify horizontal pleiotropic outliers. MR Egger: MR method which is able to assess if genetic variants have pleiotropic effects (directional pleiotropy) and provides a consistent estimate of the causal effect, under a weaker assumption called the InSIDE (INstrument Strength Independent of Direct Effect) assumption. All p-values were obtained from two-sided association tests (t-statistics).

Supplementary Figure 13. Mendelian randomization effect-effect plots on thyroid traits and non-medullary thyroid cancer

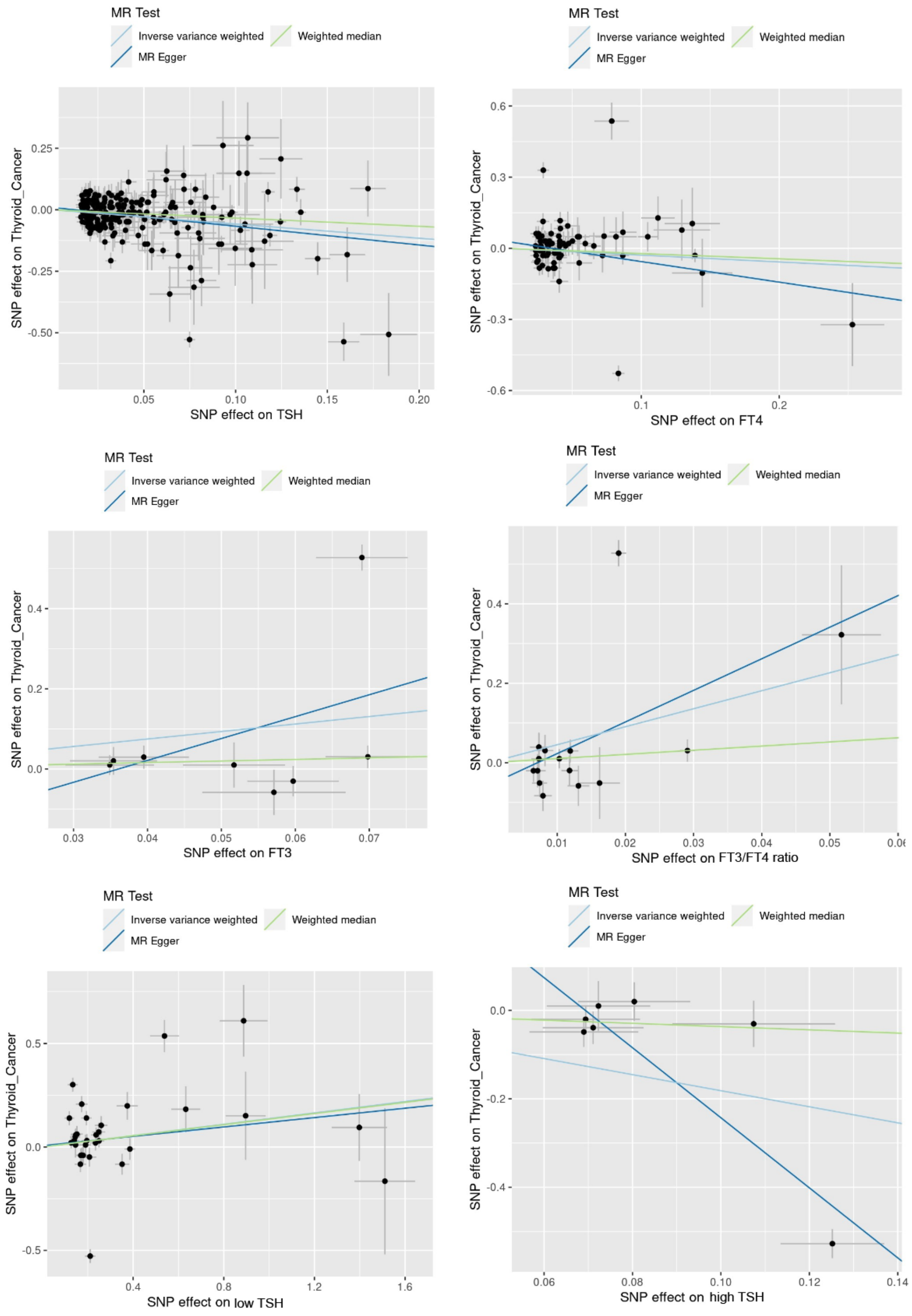

Supplement: Supplementary file 1 — Supplementary Information [file 41467_2024_44701_MOESM1_ESM.pdf]
