# Supplementary material for: Multi-trait analysis characterizes the genetics of thyroid function and identifies causal associations with clinical implications
Source: Nat Commun. 2024 Jan 30;15:888. doi: 10.1038/s41467-024-44701-9 (PMC10828500; doi:10.1038/s41467-024-44701-9)
Supplement: Supplementary file 3 — Description of Additional Supplementary Files [file 41467_2024_44701_MOESM3_ESM.pdf]

Description of Additional Supplementary Files

File Name: Supplementary Data 1
Description: Study population characteristics

File Name: Supplementary Data 2
Description: Genome-wide significant variants for reference range TSH

File Name: Supplementary Data 3
Description: Genome-wide significant variants for FT4

File Name: Supplementary Data 4
Description: Genome-wide significant variants for FT3

File Name: Supplementary Data 5
Description: Genome-wide significant variants for TT3

File Name: Supplementary Data 6
Description: Genome-wide significant variants for FT3/FT4 ratio

File Name: Supplementary Data 7
Description: Genome-wide significant variants for TT3/FT4 ratio

File Name: Supplementary Data 8
Description: Genome-wide significant variants for high TSH

File Name: Supplementary Data 9
Description: Genome-wide significant variants for low TSH

File Name: Supplementary Data 10
Description: Description of all identified thyroid function parameter loci

File Name: Supplementary Data 11
Description: Look-up of reference range TSH and FT4 index variants in previous reference range thyroid function GWAS (Teumer et al 2018)

File Name: Supplementary Data 12
Description: Genome-wide significant variants from GCTA analyses

File Name: Supplementary Data 13
Description: Summary statistics of 95% credible set SNPs in the SuSiE fine-mapping of the thyroid function traits loci

File Name: Supplementary Data 14
Description: Colocalization of index variants with mRNA expression data (GTEx)

File Name: Supplementary Data 15
Description: DEPICT results used as input for pathway analysis

File Name: Supplementary Data 16
Description: Top 10 significantly enriched canonical pathways with corresponding genes for TSH, FT4, FT3/FT4 ratio and low TSH

File Name: Supplementary Data 17
Description: Heritability enrichment analysis of thyroid function traits using thyroid scRNA-seq data

File Name: Supplementary Data 18
Description: Characteristics of GWAS meta-analyses used for the Mendelian Randomization analyses on clinical endpoints

File Name: Supplementary Data 19
Description: Association results with the outcome for the final variants included in the MR

File Name: Supplementary Data 20
Description: Mendelian Randomization of thyroid related clinical endpoints

File Name: Supplementary Data 21
Description: PheWAS results in UKBB passing Bonferroni correction using hospital acquired (HES) data for all thyroid traits.

File Name: Supplementary Data 22
Description: Polygenic risk score analysis in thyroid cancer patients (deCODE) for thyroid traits with n>5 genome-wide variants

File Name: Supplementary Data 23
Description: Variants used as instruments for Mendelian Randomization analyses with thyroid function parameters as exposure.

File Name: Supplementary Data 24
Description: Results of Mendelian Randomization (MR) analyses investigating the association between variants of thyroid function and thyroid cancer using various MR methods

File Name: Supplementary Data 25
Description: Look-up of TSH, increased TSH and decreased TSH genome wide variants in autoimmune thyroid disease GWAS (Saevarsdottir et al. 2020).
