# Supplementary material for: Multi-trait analysis characterizes the genetics of thyroid function and identifies causal associations with clinical implications
Source: Nat Commun. 2024 Jan 30;15:888. doi: 10.1038/s41467-024-44701-9 (PMC10828500; doi:10.1038/s41467-024-44701-9)
Supplement: Supplementary file 5 — Reporting Summary [file 41467_2024_44701_MOESM5_ESM.pdf]

nature portfolio

Corresponding author(s): Marco Medici, Alexander Teumer

Last updated by author(s): Nov 28, 2029

Reporting Summary

Nature Portfolio wishes to improve the reproducibility of the work that we publish. This form provides structure for consistency and transparency in reporting. For further information on Nature Portfolio policies, see our Editorial Policies and the Editorial Policy Checklist.

Statistics

For all statistical analyses, confirm that the following items are present in the figure legend, table legend, main text, or Methods section.

n/a	Confirmed	
☐	☒	The exact sample size (*n*) for each experimental group/condition, given as a discrete number and unit of measurement
☐	☒	A statement on whether measurements were taken from distinct samples or whether the same sample was measured repeatedly
☐	☒	The statistical test(s) used AND whether they are one- or two-sided<br>*Only common tests should be described solely by name; describe more complex techniques in the Methods section.*
☐	☒	A description of all covariates tested
☐	☒	A description of any assumptions or corrections, such as tests of normality and adjustment for multiple comparisons
☐	☒	A full description of the statistical parameters including central tendency (e.g. means) or other basic estimates (e.g. regression coefficient) AND variation (e.g. standard deviation) or associated estimates of uncertainty (e.g. confidence intervals)
☐	☒	For null hypothesis testing, the test statistic (e.g. *F*, *t*, *r*) with confidence intervals, effect sizes, degrees of freedom and *P* value noted<br>*Give P values as exact values whenever suitable.*
☐	☒	For Bayesian analysis, information on the choice of priors and Markov chain Monte Carlo settings
☒	☐	For hierarchical and complex designs, identification of the appropriate level for tests and full reporting of outcomes
☒	☐	Estimates of effect sizes (e.g. Cohen's *d*, Pearson's *r*), indicating how they were calculated

Our web collection on statistics for biologists contains articles on many of the points above.

Software and code

Policy information about availability of computer code

Data collection	No software for data collection for this study was used.
Data analysis	Unless stated otherwise, GWAS QC, post processing and analyses were implemented in linux shell, perl v5, python v2.7, and R v3.6 using the packages gtx (https://github.com/tobyjohnson/gtx), ggplot2, EasyQC, and TwoSampleMR. Additional analysis software used includes LD Score Regression (https://github.com/bulik/ldsc), FUMA (https://fuma.ctglab.nl/), METAL (www.sph.umich.edu/csg/abecasis/metal/), PLINK (https://www.cog-genomics.org/plink2/), DEPICT (https://github.com/perslab/depict), IPA (https://digitalinsights.qiagen.com/IPA), and GCTA (https://yanglab.westlake.edu.cn/software/gcta/).

For manuscripts utilizing custom algorithms or software that are central to the research but not yet described in published literature, software must be made available to editors and reviewers. We strongly encourage code deposition in a community repository (e.g. GitHub). See the Nature Portfolio guidelines for submitting code & software for further information.

Data

Policy information about availability of data

All manuscripts must include a data availability statement. This statement should provide the following information, where applicable:

- Accession codes, unique identifiers, or web links for publicly available datasets
- A description of any restrictions on data availability
- For clinical datasets or third party data, please ensure that the statement adheres to our policy

The individual participant data included in this project are generally not publicly available due to data privacy laws, but can be applied from the individual studies on reasonable request. We reused publically available data from the GTEx Project version 8 release (https://gtexportal.org/), the GEO accession number GSE182416 (https://www.ncbi.nlm.nih.gov/geo/query/acc.cgi?acc=GSE182416), as well as summary statistics of the following GWAS: body mass index, height, waist-hip ratio, heart rate (resting state), blood pressure (systolic/diastolic), pulse pressure, lipids (LDL, HDL, total cholesterol, triglycerides), type 2 diabetes mellitus, atrial fibrillation, coronary artery disease, heart failure, stroke, bone mineral density, fractures, muscle weakness, Intelligence Quotient, Alzheimer's disease, major depressive disorder, anxiety, bipolar disorder, thyroid cancer, and thyroid function (TSH and FT4), with corresponding references for access provided in the Methods section. The data of the UK Biobank can be applied via the study website (https://www.ukbiobank.ac.uk/). The summary statistics from the GWAS meta-analyses as well as the complete colocalization results and regional association plots of the fine-mapping analyses generated in this project are available on the ThyroidOmics Consortium website (http://www.thyroidomics.com) at the Datasets section (https://transfer.sysepi.medizin.uni-greifswald.de/thyroidomics/datasets/). Source data are provided with this paper.

Research involving human participants, their data, or biological material

Policy information about studies with human participants or human data. See also policy information about sex, gender (identity/presentation), and sexual orientation and race, ethnicity and racism.

Reporting on sex and gender	Sex was determined on self-reported data and/or genetic information. Sex-combined analyses were performed, and the association models were adjusted for sex as a covariate. No individual level data has been shared.
Reporting on race, ethnicity, or other socially relevant groupings	Individuals of European ancestry were included in this project. Ethnicity was self-reported or estimated based on the genetic profile.
Population characteristics	In this project, predominantly population-based studies were included. Details of each cohort are provided in the Supplementary Table 1.
Recruitment	No participants were recruited specifically for this projects. Recruitment of the participants of the individual studies included in this projects are described in the respective study specific publications listed in the Supplementary Note and Supplementary Table 1.
Ethics oversight	All participants provided written informed consent, and each participating study was approved by the respective ethics committe: the ethical committees for Frederiksberg and Copenhagen, the University of Maryland Institutional Review Board, the Institutional Review Board of each ARIC site, the University of Western Australia Human Research Ethics Committee, the Internal Review Board of the National Institute for Environmental Health Sciences, the Human Research Ethics committee of the Queensland Institute of Medical Research, the coordinating center at the University of Washington in Seattle, the Ethics Committee of the Healthcare System of the Autonomous Province of Bolzano/Bozen, the University of Split School of Medicine, the Norfolk Research Ethics Committee, the National Health Service (NHS) Health Research Authority Research Ethics Committee, the Boston Medical Center, the medical ethics committee of the Leiden University Medical Center, the local ethic committees of the GCKD study and registered in the national registry for clinical studies, the Danish Data Protection Agency, the Tayside Medical Ethics Committee, the Institutional Review Board of the National Public Health Institute (Helsinki, Finland), the Ethics Committee of the Copenhagen Region, the Norwegian Data Protection Authority and the Regional Committee for Medical and Health Research Ethics in Central Norway, the Italian National Institute of Research and Care of Aging Institutional Review and by the Internal Review Board of the National Institute for Environmental Health Sciences (NIEHS), the local ethical committee of the Inter99 study, the ethics committee of the Bavarian Medical Association, the Lothian Research Ethics Committee, the medical ethical committee of the Leiden University Medical Center, the medical ethical committee of the University Medical Center Groningen, the Institutional Review Board at the University of Michigan, the Radboud university medical center Institutional Review Board, the institutional ethics review boards of centers of Cork University (Ireland), Glasgow University (Scotland) and Leiden University Medical Center (the Netherlands), the MyCode Governing Board, the Medical Ethics Committee of the Erasmus MC, the institutional review boards for the Istituto di Neurogenetica e Neurofarmacologia, for the MedStar Research Institute and for the University of Michigan, the medical ethics committee of the University of Greifswald, the ethical committee of the Ghent University Hospital, the National Research Ethics Service London-Westminster, the St Thomas' Hospital Research Ethics Committee, the Ethics Committee of the San Raffaele Hospital and of the Piemonte Region, local IRB of the Women's Genome Health Study, the North West Centre for Research Ethics Committee, the Icelandic National Bioethics Committee.

Note that full information on the approval of the study protocol must also be provided in the manuscript.

Field-specific reporting

Please select the one below that is the best fit for your research. If you are not sure, read the appropriate sections before making your selection.

☒ Life sciences ☐ Behavioural & social sciences ☐ Ecological, evolutionary & environmental sciences

For a reference copy of the document with all sections, see nature.com/documents/nr-reporting-summary-flat.pdf

Life sciences study design

All studies must disclose on these points even when the disclosure is negative.

Sample size	Discovery project using maximum sample size available effectively doubling sample size of former projects. Therefore, no sample-size calculation was performed.
Data exclusions	Participants aged <18 years, of non-European ancestry, using thyroid medication (defined as ATC code H03), or with a history of thyroid surgery were excluded from all analyses. Only studies having at least 40 cases were considered in the high and low TSH GWAS analyses.
Replication	Replication of the GWAS results could not be conducted because no additional independent samples were available. However, we validated the TSH and FT4 association results once with the results of the formerly published study of Teumer et al. (2018).
Randomization	Samples were population based. The analyses were adjusted for age, sex and relevant study-specific covariates such as principal components for population stratification, study center and family-structure (e.g. by inclusion of the kinship matrix as a random effect) if applicable.
Blinding	Cohort allocation was not part of this project and was performed accordingly prior to this project.

Reporting for specific materials, systems and methods

We require information from authors about some types of materials, experimental systems and methods used in many studies. Here, indicate whether each material, system or method listed is relevant to your study. If you are not sure if a list item applies to your research, read the appropriate section before selecting a response.

Materials & experimental systems

n/a	Involved in the study	
☒	☐	Antibodies
☒	☐	Eukaryotic cell lines
☒	☐	Palaeontology and archaeology
☒	☐	Animals and other organisms
☒	☐	Clinical data
☒	☐	Dual use research of concern
☒	☐	Plants

Methods

n/a	Involved in the study	
☒	☐	ChIP-seq
☒	☐	Flow cytometry
☒	☐	MRI-based neuroimaging
